# Temporal responses of conserved miRNAs to drought and their associations with drought tolerance and productivity in rice

**DOI:** 10.1186/s12864-020-6646-5

**Published:** 2020-03-14

**Authors:** Hui Xia, Shunwu Yu, Deyan Kong, Jie Xiong, Xiaosong Ma, Liang Chen, Lijun Luo

**Affiliations:** Shanghai Agrobiological Gene Center, Shanghai, China

**Keywords:** microRNA, Transcriptome, Posttranscriptional regulation, Drought-tolerance, Breeding, *Oryza sativa*

## Abstract

**Background:**

Plant miRNAs play crucial roles in responses to drought and developmental processes. It is essential to understand the association of miRNAs with drought-tolerance (DT), as well as their impacts on growth, development, and reproduction (GDP). This will facilitate our utilization of rice miRNAs in breeding.

**Results:**

In this study, we investigated the time course of miRNA responses to a long-term drought among six rice genotypes by high-throughput sequencing. In total, 354 conserved miRNAs were drought responsive, representing obvious genotype- and stage-dependent patterns. The drought-responsive miRNAs (DRMs) formed complex regulatory network via their coexpression and direct/indirect impacts on the rice transcriptome. Based on correlation analyses, 211 DRMs were predicted to be associated with DT and/or GDP. Noticeably, 14.2% DRMs were inversely correlated with DT and GDP. In addition, 9 pairs of mature miRNAs, each derived from the same pre-miRNAs, were predicted to have opposite roles in regulating DT and GDP. This suggests a potential yield penalty if an inappropriate miRNA/pre-miRNA is utilized. miRNAs have profound impacts on the rice transcriptome reflected by great number of correlated drought-responsive genes. By regulating these genes, a miRNA could activate diverse biological processes and metabolic pathways to adapt to drought and have an influence on its GDP.

**Conclusion:**

Based on the temporal pattern of miRNAs in response to drought, we have described the complex network between DRMs. Potential associations of DRMs with DT and/or GDP were disclosed. This knowledge provides valuable information for a better understanding in the roles of miRNAs play in rice DT and/or GDP, which can facilitate our utilization of miRNA in breeding.

## Background

MicroRNAs (miRNAs) are a large class of small noncoding RNAs of 20 to 24 nucleotides (nt) in length [36, 43, 44]. The miRNA and its target mRNA can form the miRNA-induced silencing RISC complex, which inhibits the protein of its target genes by either destabilizing the mRNA or by inhibiting its translation [43, 44]. The RISC complex negatively regulates gene expression at the posttranscriptional level. miRNA target transcription factors, many of which are critical regulators in plant growth, development, and reproduction (GDP), and stress responses [36, 38, 49]. Therefore, a miRNA that has great impacts on the transcriptome, is located at the center of complex gene regulatory networks associated with plant GDP and stress-tolerance, [7, 38]. The ability of plants to employ miRNAs to posttranscriptionally inactive or induce the expression of stress-responsive genes provides an advantage compared with regulation by transcription factors alone [49]. It makes miRNAs good targets to improve crop stress-tolerance [7, 38, 49]. However, most miRNAs do not work independently in response to environmental stresses. Their stress responses are tightly coordinated with multiple developmental processes via the complex regulatory network [7, 38] or multihormone responses [29]. Increasing evidence has shown that a miRNA involved in stress tolerance commonly exerts pleiotropic effects on the GDP of plants [38]. It means we should avoid potential negative effects on productivity when developing tolerant cultivars by genetically modifying miRNAs. This requires improved understanding of the association of a miRNA with stress-tolerance and/or GDP.

Drought is a major limiting environmental factor for crops and causes great loss in yield annually. It is essential to develop drought-tolerant crops for food security [12]. Recently, attentions have been focused on the importance of posttranscriptional regulation by miRNAs in drought tolerance (DT) due to their central roles in the regulatory network [7, 38]. With the fast development of next-generation sequencing, drought-responsive miRNAs (DRMs) have been identified in diverse crops, including cotton [48], rapeseed [21], maize [1], tomato [28], and rice (*Oryza sativa*) [3, 5, 6]. Many DRMs have been characterized as important modulators in DT via regulating the expression of drought-responsive genes [7]. Most miRNAs are induced by drought and downregulate their target mRNAs [7], which are negative factors in the drought response [8, 53]. Conversely, some other miRNAs are downregulated by drought [7], leading to the accumulation of target mRNAs positively contributing to drought adaptation [25, 37].

Rice is one of the most important cereal food for more than half of the global population. Unfortunately, elite rice is very sensitive to drought due to its long-term domestication in irrigated fields [4, 45]. The improvement of DT in rice is thus a primary breeding aim for “green super rice” [31, 52]. For this purpose, the roles played by miRNAs in rice drought-resistance have been widely studied. There have been 604 pre-miRNAs, which encode 738 mature miRNAs, identified in rice and recorded in miRBase (release 22.1, [22]). Hundreds of miRNAs have been determined as DRMs by several genome-wide investigations in different genotypes or tissues [2, 3, 6, 56]. However, there is still a large knowledge gap between the identification of DRMs and the characterization of their associations with DT [17]. According to large number of recommended DRMs, only very low proportions of DRMs have been functionally proven in rice, including miRNA162 [40], miRNA164 [8], miRNA166 [51], miRNA393 [46], and miRNA408 [37]. Among these drought-tolerant miRNAs, miRNA408 [37, 50] and miRNA393 [46] have been reported to have unwanted pleiotropic effects on GDP. For better utilization of miRNAs, it is necessary to understand their associations with DT and/or GDP in rice.

Many former studies have typically investigated a single genotype [3, 6, 56] or two rice genotypes of contrasting DT [5] to identify DRMs. A miRNA that is differentially regulated in response to environmental stress is not necessarily associated with stress tolerance [17]. Therefore, it is essential to study diverse genotypes, which allows us to eliminate bias caused by a limited number of genotypes. Rice adaptation to drought is a progressive process with sequential molecular, physiological, and morphological alterations [9, 35]. However, the time course of miRNA expression and regulations in rice under drought has not been fully understood, but from it we can learn potential associations between miRNAs and physiological/ morphological responses [17]. To understand the potential roles played by miRNAs in rice DT, we investigated the genome-wide expression of miRNAs in six rice genotypes at five time points under drought stress and one time point at recovery. Meanwhile, we also investigate the transcriptomes of six genotypes by RNA-sequencing, from which we can learn the potential impacts of miRNAs on the rice transcriptome. The design of our experiment allows us to address the following questions: (1) How are miRNAs sequentially regulated in response to progressive drought? (2) Do any DRMs associate with drought-tolerance and/or GDP? (3) Which miRNAs are good candidates for improving rice DT? This knowledge can advance our utilization of miRNAs to improve DT without yield penalty in rice.

## Results

### Alterations of morphological and physiological traits among rice genotypes under drought conditions

**Fig. 1:**
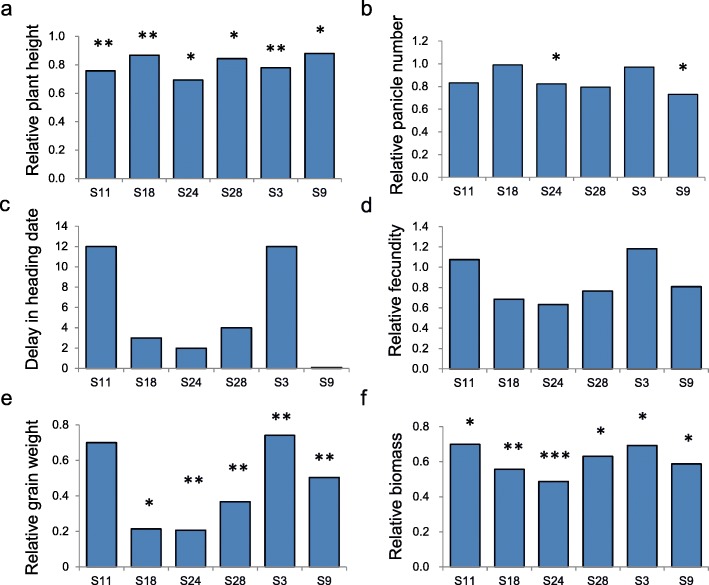
Relative performances (performance under drought (DT) /that under well-watered (CK)) of six rice genotypes. * indicates significant differences between traits measured in DT and those measured in CK

The growth, development, and productivity of six rice genotypes were greatly affected by drought, as reflected in reduced plant height, number of seeds per plant, seed weight per plant, and biomass, and delayed heading date (Fig. 1, Additional file: Table S1). Drought also caused the accumulation of H_2_O_2_ content (Additional file: Table S2) and dead leaves (Additional file: Table S1) in the rice genotypes. To resist drought, the rice activated mechanisms of osmotic adjustment and ROS scavenging, as reflected in the largely increased osmotic potential (Additional file: Table S3) and total antioxidant capacity (Additional file 1: Table S4) under drought conditions, particularly in later drought time points (D3-D5).

### Sequence analysis of small RNAs in sequenced samples

A total of ~ 1.068G raw reads were obtained from 66 samples (libraries). After the removal of low-quality reads, adapters, reads shorter than 18 nt, and other contaminating sequences, 792.8 M clean reads (74.3%) were finally retained, including 172.4 M unique reads (Additional file 1: Table S5). Among total clean reads between 18 and 32 nt, 39.5% reads were matched to miRNA (~ 21%), tRNA (~ 5%), and rRNA (~ 12%), respectively (Additional file 2: Figure S1). The distribution of reads in various sizes of small RNAs was not homogeneous. The most abundant were small RNAs of 21 nt (27.4%) and 24 nt (20.1%) in length (Additional file 2: Figure S2). We should also point out that proportions of miRNAs of 21 nt and 24 nt in length had great variations among genotypes, time points, and treatments (Additional file 2: Figure S2).

### General description of drought-responsive and recovery-related miRNAs detected in the six rice genotypes

A total of 632 conserved mature miRNAs in miRBase were detected in 66 sequenced samples (Additional file 1: Table S6). Among the expressed miRNAs, 549 miRNAs were available for further analysis (TPM > 0.1 in at least one sample) (Additional file 1: Table S6). During the drought period, 354 miRNAs in 57 families were identified as drought-responsive miRNAs (DRMs). Moreover, 80 differentially expressed miRNAs were detected at the recovery stage and were determined to be recovery-related miRNAs (RRMs) (Additional file 1: Table S6). A considerable proportion (48.6%, 172 out of 354) of DRMs were regulated in a genotype-specific (Additional file 2: Figure S3) or temporal-specific (Additional file 2: Figure S4) manner. There were 78–239 DRMs and 77–116 RRMs among the six genotypes (Table S6). A susceptible genotype S18 (239) and a tolerant genotype S11 (216) had the most DRMs. Meanwhile, a susceptible genotype S24 (78) and a tolerant genotype S28 (87) had the least DRMs. This result indicated the number of DRMs should be not related with rice drought tolerance. However, 107 DRMs could be frequently (frequency ≥ 3) detected among different genotypes and time points (Additional file 2: Figure S5a), suggesting that they have universal roles in rice adaptation to drought. We also detected great variance in number of RRMs among the six genotypes. Interestingly, the three tolerant genotypes S3, S11, and S28 possessed more RRMs (from 6 to 66), while the susceptible ones had less RRMs. Finally, we detected no recovery-specific differentially expressed miRNAs (Additional file 2: Figure S5b). In addition, regulation patterns of most characterized miRNAs (e.g. miR160, miR162, miR393, miR397, and miR408) were consistent with previous studies (Additional file 1: Table S6). However, regulations of some other characterized miRNAs (e.g. miR166, miR172, and miR396) represented great variation among genotypes (Additional file 1: Table S6).

### Correlations of expressions among DRMs

**Fig. 2:**
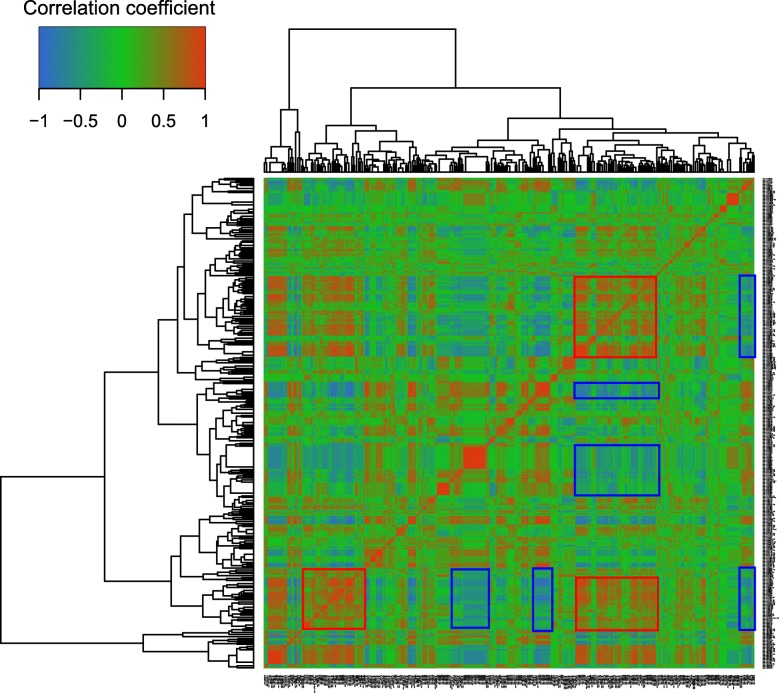
A heatmap of the matrix of Pearson’s correlation coefficient among drought-responsive miRNAs based on their expressions. Red and blue frames represent some examples of significantly positive and negative correlations among miRNAs

Coexpression relationships between DRMs were revealed by their positive or negative correlations (Fig. 2). Pearson correlation coefficients (PCCs) (0.620 ± 0.013, *p* < 0.001) between miRNAs of the same family (e.g., miRNA169, miRNA395, miRNA818) or PCCs between pairs of miRNAs derived from the same pre-miRNAs (0.453 ± 0.059, *p* < 0.001) (e.g., miRNA1320-3p/5p, miRNA528-3p/5p, miRNA7695-3p/5p) were significantly higher than the average PCC (0.084 ± 0.007) by both Mann-Whitney and Kolmogorov-Smirnov tests. High PPC values could also be frequently detected between some unrelated miRNAs (e.g. miRNA1862 with miRNA169 and miRNA869, miRNA156 with miRNA169 and miRNA815) (Fig. 2). Additionally, we detected many negatively correlated miRNAs (e.g., miRNA818 with miRNA169 and miRNA166, miRNA395 with miRNA169). These results indicated complicated regulatory networks of miRNAs in response to drought.

### Correlations of miRNAs with GDP and DT traits

**Fig. 3:**
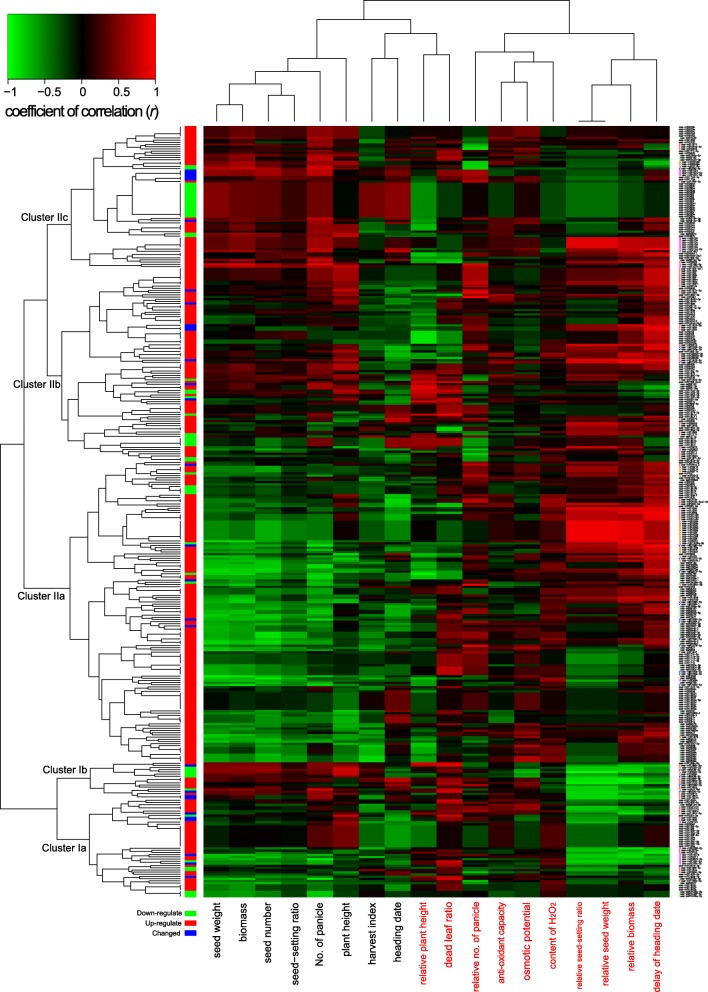
A heatmap of Pearson’s correlation coefficient (PCC) between drought-responsive miRNAs (DRMs) and agronomic (in blue) and drought-tolerant (DT) (in red) traits. Five types of DRMs are at right: Type I, a miRNA is significantly correlated (|PCC| > 0.6) with at least one of measure agronomic traits; Type II, a miRNA is significantly correlated (|PCC| > 0.6) with at least one of measured DT traits; Type III, a miRNA is positively or negatively correlated with both agronomic and DT traits; Type IV, a miRNA is oppositely correlated (|PCC| > 0.6) with measured agronomic and DT traits; Type V, a pair of miRNAs are oppositely correlated (|PCC| > 0.6) with measured agronomic and DT traits

Based on their correlations with GDP and DT, miRNAs could be generally grouped into five clusters (Fig. 3). Cluster Ib contained 39 miRNAs. Their expression levels were generally positively correlated with GDP traits, while their expression/regulation levels were negatively correlated with DT traits. Cluster IIa contained 138 miRNAs. Their expression levels were generally negatively correlated with GDP traits, while their expression/regulation levels were positively correlated with DT traits (Fig. 3). miRNAs in cluster Ib and IIa played opposite roles in regulating GDP and DT. Only a few miRNAs were both positively/negatively correlated with GDP and DT traits (mainly distributed in cluster Ia and cluster IIc) (Fig. 3).

Four types of miRNA could be further defined by their correlations with GDP and/or DT using threshold of |PCC| ≥ 0.6. There were 74, 68, 21, and 30 miRNAs classified into type I, II, III, and IV, respectively (Fig. 3). The prediction based on the correlation analysis was partially validated by the miRNAs that have been functionally characterized in rice (Additional file 1: Table S7) [14–16, 20, 24, 27, 47, 54, 57]. The regulation of a miRNA in response to drought always tended to enhance DT and had negative impacts on GDP (Table 1). Interestingly, DRMs of the same type were more generally highly correlated (Fig. 2, Additional file 1: Table S8). In addition, DRMs of different types, which played similar roles in DT and/or GDP, possessed higher mean PPCs. For example, the mean PPCs among types I-b, III-b, and IV-b, which were positively correlated with GDP traits, ranged from 0.254~0.335 (Additional file 1: Table S8). Similarly, the mean PPCs between types II-b and III-b, which tended to increase DT, were as high as 0.176. Above results indicated DRMs with similar functions worked together to resist drought (Additional file 1: Table S8).

**Table 1:** miRNAs of different types in responses to drought

Type	Correlation with GDP	Correlation with DT	No. of miRNA	Upregulation	Ratio	Downregulation	Ratio	Varied among genotypes
I-a	−1	0	69	61	0.884	5	0.072	3
I-b	1	0	12	8	0.667	2	0.167	2
II-a	0	-1	29	16	0.552	5	0.172	8
II-b	0	1	44	37	0.841	1	0.023	6
III-a	-1	-1	8	5	0.625	1	0.125	2
III-b	1	1	13	8	0.615	2	0.154	3
IV-a	-1	1	27	25	0.926	1	0.037	1
IV-b	1	-1	8	2	0.250	6	0.750	0
Overall	–	–	354	267	0.754	54	0.153	33

The miRNAs of type V were allocated in to type I~IV based on their correlations with DT and/or GDP

“1” indicates positive correlations (PPC > 0.60); “− 1” indicates negative correlations (PPC < -0.60); “0” indicates no correlation

*PCC* Pearson correlation coefficient, *GDP* growth, development, and reproduction, *DT* drought tolerance

We also noticed that a pair of mature miRNAs derived from the same pre-miRNA may sometimes have opposite and independent impacts on GDP and DR. In this study, nine pairs of mature miRNAs derived from the same pre-miRNA demonstrated this pattern (defined as type V) (Fig. 3). For example, *OsmiR1870-3p* and *OsmiR1870-5p*, were derived from the same stem-loop structure of *pre-OsmiR1870*. The expressions of *OsmiR1870-3p* and *OsmiR1870-5p* were not correlated (PCC = 0.141, *p* > 0.05) (Fig. 2). The expression of *OsmiR1870-3p* was negatively correlated with plant height (PCC = -0.802) and biomass (PCC = -0.664) (Fig. 3), indicating a negative role in regulating rice growth and productivity. The expression of *OsmiR1870-5p* were positively correlated with AOC (PCC = 0.602), relative seed-setting ratio (PCC = 0.826), relative seed weight (PCC = 0.826), and relative biomass (PCC = 0.996) (Fig. 3), indicating its positive role in rice DT.

### Time course of the regulation of miRNAs

**Fig. 4:**
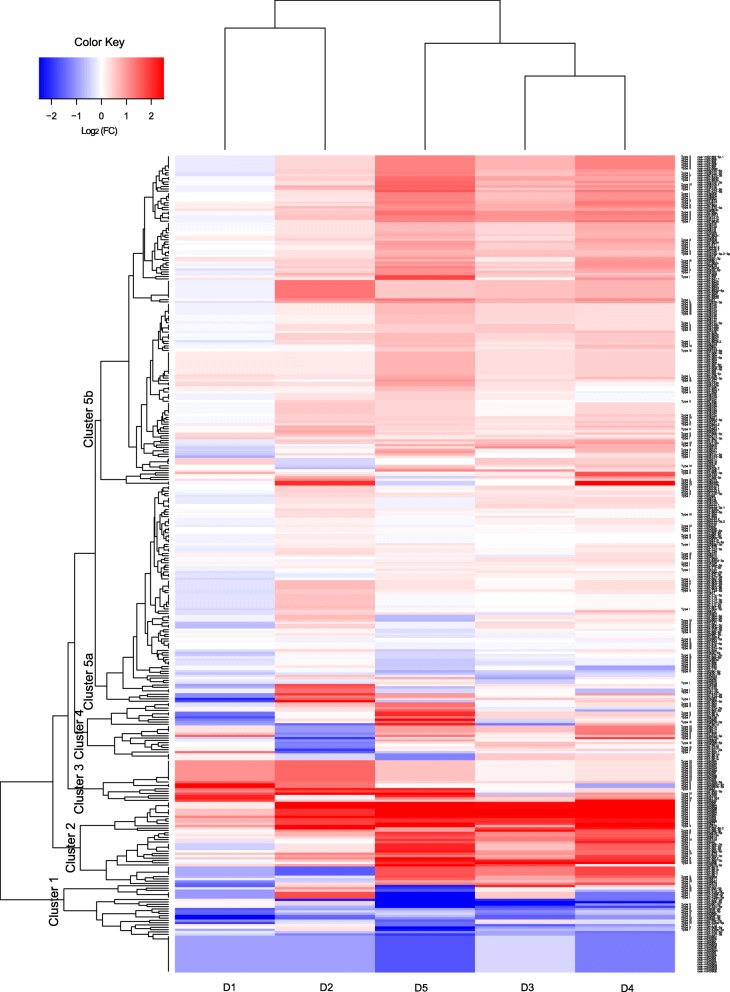
A heatmap of time-series regulations of drought-responsive miRNAs (DRMs) during drought period. The regulation of a DRM is quantified by Log_2_ (its expression under drought/ its expression under well-watered condition). Five major clusters (1~5) are generated by hierarchical clustering (Euclidean method)

Based on the regulation of miRNA expression in response to drought (log_2_FC), 354 DRMs formed five major time course clusters (Fig. 4). Cluster− 1 contained 37 DRMs (5, 3, 3, and 3 for types I, II, III, and IV, respectively), which were gradually downregulated throughout the progress of drought. Cluster-2 also contained 37 DRMs (18, 5, 2, and 3 for types I, II, III, and IV, respectively), which were highly upregulated starting at time point D2, particularly at the later drought time points (D3-D5). This indicated that DRMs in cluster-2 might be associated with DT in the late stage. Cluster-3 contained 18 DRMs (1, 4, 0, and 11 for types I, II, III, and IV, respectively), which were significantly upregulated at the early drought time points (D1 and D2). This indicated that DRMs in cluster-2 might be associated with DT in the early stage. Cluster-4 contained 28 DRMs (2, 8, 3, and 3 for types I, II, III, and IV, respectively), which had significant changes in expression (upregulation and/or downregulation) at one or two time points. Cluster-5, which could be further divided into two sub-clusters (5a and 5b), contained 234 DRMs. In particular, 143 DRMs (31, 30, 8, and 7 for types I, II, III, and IV, respectively) in cluster-5b exhibited greater fold changes than DRMs in the cluster-5a and were gradually upregulated throughout the period of drought, indicating that they have important roles in rice DT.

At the recovery stage, 80 RRMs could be divided into 5 types based on their regulatory patterns from the D5 to R stages (Additional file 1: Table S9). In patterns A and B, 52 RRMs maintained their upregulation and downregulation at recovery (Additional file 1: Table S9). In patterns C and D, 24 RRMs had recovered from their regulatory patterns during drought conditions (Additional file 1: Table S9). The regulatory patterns of another four RRMs were varied among the six genotypes (coded as Type E).

### Impacts of DRMs and RRMs on the rice transcriptome

Predicted by both TargetFinder and psRobot, 195 DRMs had 612 target genes in total, which contained 202 DRGs (Additional file 1: Table S10). Many of these target DRGs were characterized as with GDP- and/or DT-associated genes (Additional file 1: Table S10). Noticeably, 35.3% (166 out of 470) and 18.1% (85 out of 470) predicted target DRGs were significantly (PCC > 0.242, *p* < 0.05) and moderately (|PCC| > 0.400) correlated with its regulatory miRNAs (Additional file 1: Table S11). This result indicated that correlations between miRNA and DRGs among genotypes can provide important cues to determine the candidate target gene for a DRM. For example, expressions of a predicted target gene *LOC_Os12g40890* was positively correlated (PCC = 0.464) with its regulatory *osa-miR408-5p* (Additional file 1: Table S12). As expected, the expression of *LOC_Os12g40890* was increased in the overexpression lines of *pre-OsmiR408* (Additional file 2: Figure S6), which potentially regulated DT in the transgenic lines. However, we still noticed that a proportion of predicted target genes were not correlated with its regulatory miRNA among genotypes. For example, expressions of *OsUCL8* (*LOC_Os03g50140*) and *LOC_Os08g37670*, which have been verified as the target of *osa-miR408-3p* [37, 50], was not correlated with *osa-miR408-3p* among genotypes (Additional file 1: Table S12). It means that target genes were not always significantly correlated with its regulatory miRNA among genotypes.

Besides the direct regulation on target genes, miRNAs could also have profound indirect influences on the rice transcriptome, as revealed by the large number of correlated DRGs. A DRM was highly (|PCC| > 0.6) and moderately (|PCC| > 0.4) correlated with ~ 90.0 (ranging from 0 to 1630) and 868.4 (ranging from 26 to 2895) DRGs on average (Additional file 1: Table S11; Additional file 3). Based on their highly correlated DRGs, these DRMs and RRMs were involved in diverse GO biological processes (Additional file 2: Figure S7) and KEGG pathways (Additional file 2: Figure S8).

**Fig. 5:**
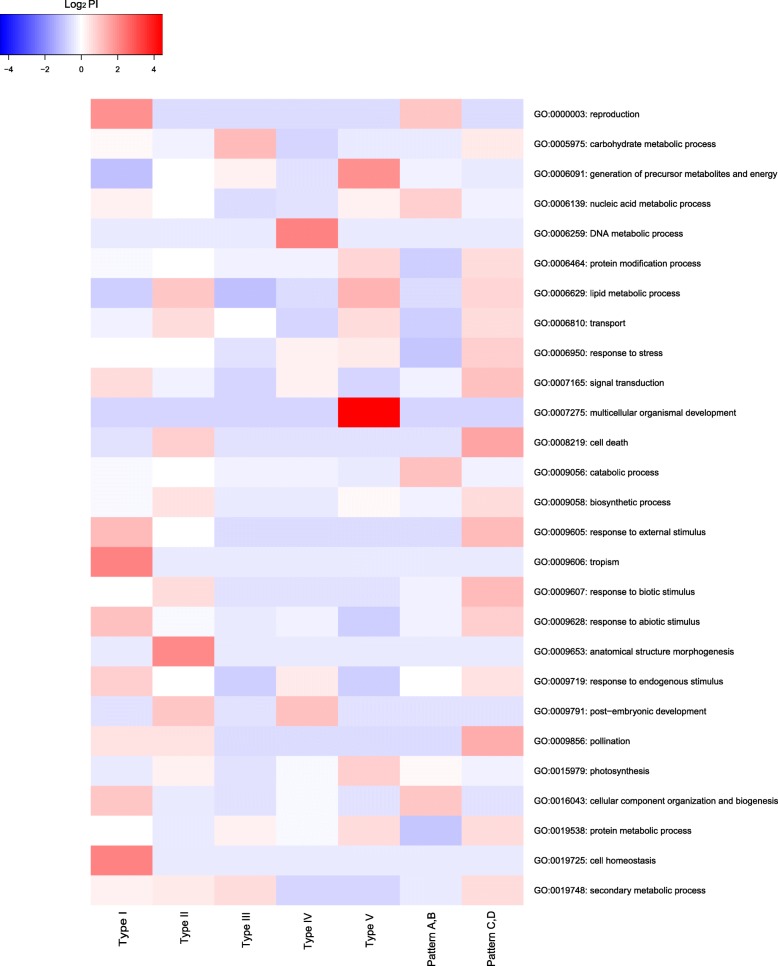
A heatmap of preferential index (PI) of five types of drought-responsive miRNAs (DRMs) and two patterns of recovery-related miRNAs (RRMs) in the categorized Gene Ontology (GO) biological processes. A high PI indicates the type of DRMs or the pattern of RRMs preferentially anticipates the certain biological process

Different types of DRMs had their preferential categories of GOBPs (Fig. 5) and KEGG pathways (Additional file 2: Figure S8). For example, DRMs of type I tended to be involved in reproduction, cellular component organization and biogenesis, cell homeostasis, response to external stimulus, response to abiotic stimulus, and response to endogenous stimulus. DRMs of type II tended to be involved in the generation of precursor metabolites and energy, photosynthesis, lipid metabolic process, transport, cell death, biosynthetic process, anatomical structure morphogenesis, and post-embryonic development. DRMs of type III tended to be involved in carbohydrate metabolic process, generation of precursor metabolites and energy, protein metabolic process, and secondary metabolic process. DRMs of type IV tended to be involved in DNA metabolic process and post-embryonic development (Fig. 5). At the recovery stage, RRMs in patterns A and B tended to be involved in reproduction, cellular component organization and biogenesis, nucleic acid metabolic process, and catabolic process. RRMs in patterns C and D tended to be involved in carbohydrate metabolic process, protein modification process, transport, response to stress, cell death, and signal transduction (Fig. 5).

The transcriptomic impact of a miRNA and its involvement in certain biological processes can provide additional information for us to understand its role in DT and/or GDP. For instance, *OsmiR1870-3p* and *OsmiR1870-5p* were predicted to be associated with GDP and DT, respectively (Fig. 3). *OsmiR1870-3p* were correlated with 289 DRGs (absolute PCC > 0.4, *p* < 0.05), while *OsmiR1870-5p* were correlated with 1598 DRGs (absolute PCC > 0.4, *p* < 0.05) (Additional file 3, Additional file 2: Figure S9a). DRGs correlated with *OsmiR1870-3p* and *OsmiR1870-5p* were rarely overlapped (Additional file 2: Figure S9a). Among the correlated DRGs, there were many characterized genes of GDP and DT (Additional file 1: Table S13). Moreover, the result of GO enrichment indicated that *OsmiR1870-5p* were involved in response to stimuli (GO:0050896) and response to stress (GO:0006950) (Additional file 2: Figure S9c). This partially supported the predictions of *OsmiR1870-5p* was associated with DT.

### Candidate miRNAs of drought-tolerance

The DRMs from types II, III, and V, which were associated with DT but without potential adverse effects on GDP, could be valuable candidates for the improvement of rice DT. A miRNA has a high probability to be associated with DT if it has direct or indirect impacts on the expression of DT genes. Based on the above considerations, 24 DRMs in 15 families were recommended as good candidates for DT improvement. These DRMs had 2–23 moderately correlated (|PPC| > 0.4) known DT genes (Table 2, Additional file 3). They belonged to time course cluster-2 and cluster-5b, which play roles in DT at different stages. Based on their highly correlated DRGs (|PPC| > 0.6), the candidate miRNAs anticipated many biological processes (Additional file 2: Figure S7) and metabolic pathways (Additional file 2: Figure S8) relevant to DT. For example, *osa-miR399k* may enhance DT by regulating water transport (GO:0006833) and fluid transport (GO:0042044) (Additional file 2: Figure S7), while *osa-miR169* (*g* and *f.1*) may regulate DT by thiamine (ko00730) and vitamin B6 (ko00750) metabolism (Additional file 2: Figure S8).

**Table 2:** Candidate miRNAs for drought tolerance (DT). A correlated DT gene means its absolute Pearson’s correlation coefficient is > 0.4

miRNA	Type	Cluster	No. of predicted target genes^a^	No. of correlated DT genes^b^
*osa-miR159 (c, d)*	II	Cluster-5b	8	13
*osa-miR169 (f.1, g)*	II	Cluster-5b	8	9
*osa-miR169f.2*	II	Cluster-2	1	9
*osa-miR1870-5p*	V	Cluster-5b	0	18
*osa-miR2121 (a, b)*	II	Cluster-2	6	23
*osa-miR2875*	II	Cluster-2	1	7
*osa-miR398a*	II	Cluster-2	1	6
*osa-miR399k*	III	Cluster-2	3	3
*osa-miR5144-5p*	III	Cluster-5b	0	10
*osa-miR5148 (a, b, c)*	II	Cluster-5b	0	5
*osa-miR5505*	II	Cluster-5b	0	8
*osa-miR5508*	II	Cluster-5b	6	2
*osa-miR5513*	II	Cluster-5b	0	3
*osa-miR5811*	III	Cluster-2	0	15
*osa-miR812 (a, b, c, d, e)*	III	Cluster-5b	21	6

^a^Detail information of predicted target genes is in Table S8

^b^Detail information of correlated genes is in Additional file 3

## Discussion

### Time course of the regulation of miRNAs under drought conditions provides key information to identify miRNAs associated with DT

miRNAs are in the response to drought stress and play essential roles in rice DT [7, 49]. Many previous studies have described responses of miRNAs to drought in different rice genotypes [3, 5, 6] and tissues [2, 18]. The common design of these experiments is to generate drought-responsive miRNAs from a single genotype [2, 6, 56] or a pair of genotypes with contrasting tolerance [3, 5]. The involvement of a limited number of genotypes and time points when investigating DRGs may result in the loss of valuable information [17]. By time course investigation of miRNAs in response to drought among six rice genotypes, we identified greater number of conserved drought-responsive miRNAs than previous studies (354 mature miRNA in 57 families). Genotype-specific expression and regulation of a miRNA in response to drought is common [33, 49]. The expression and regulation of a miRNA could be randomly different between two genotypes. Therefore, the identification of candidate miRNAs simply by generating differentially regulated miRNAs from two genotypes with contrasting drought tolerances may lead to false-positive results. By including a considerable number of samples, we can apply a correlation analysis to build the association of a miRNA with DT/GDP. The correlation-based prediction is reliable, as it is validated by many former functional studies of miRNAs. This correlation-based prediction provides us the key information to identify miRNAs associated with DT.

We also describe time course regulations of DRMs of different types during drought periods. These results can provide additional clues to understanding the role a miRNA plays in DT and/or GDP. For instance, the DRMs in cluster− 1 and cluster-5b were regulated in a drought-dependent manner, with regulation levels that gradually increased along with drought progression. GDP-associated DRMs in this cluster were passively regulated to slow down rice growth, while DT-associated DRMs in this cluster had minor but additive impacts on DT. The DRMs in cluster-2 were largely upregulated starting at D2, indicating their crucial roles during later drought periods, such as the strong activation of osmotic adjustment. In contrast, the DRMs in cluster-3 play a role in early drought. It is noteworthy that cluster-3 contains a high proportion of DRMs that are inversely correlated with both DT and GDP (type IV) and potentially have pleiotropic effects on GDP. Their activations limited in the early drought may bring less penalties on GDP.

### Complex regulatory networks of miRNAs in rice DT and/or GDP

First, we detected a complex regulatory network of DRMs based on the correlation analysis. We found that DRMs of the same type worked together to activate the tolerant mechanisms and/or to inhibit rice GDP. Second, a miRNA could have profound impacts on the plant transcriptome [7, 38]. In our study, we observed that a large number of DRGs are highly correlated with DRMs. Which make them be involved in diverse GO biological processes and/or KEGG metabolic pathways. The involvement of DRMs in certain GO biological processes and KEGG pathways could provide additional information to understand the role of a miRNA plays in DT and/or GDP. For example, DT-associated DRMs tended to be involved in energy metabolism (e.g., photosynthesis, generation of precursor metabolites and energy). This result highlights the point that DT in a crop requires the ability to maintain normal GDP under drought conditions, rather than merely to survive [32]. The GDP-associated DRMs (miRNAs of type I) tended to be involved in stress responses (e.g., tropism, cell homeostasis, response to abiotic stimulus). This indicates that the miRNA-mediated regulation of GDP is relevant to environmental adaptation. We also detected a few (21 out of 354) miRNAs belonging to type III. They tended to be involved in carbohydrate metabolic processes (e.g., trehalose metabolic process, trehalose biosynthetic process). These metabolic or biological processes have been reported to be closely related to better productivity under drought conditions [10, 34]. Therefore, DRMs of type III are valuable candidates for improving DT.

### Tradeoff between DT and GDP by miRNAs

The activation of DT mechanisms is always coordinated with multiple developmental processes [11, 35]. Given the central role of miRNAs in the regulatory network [7, 38], a DT-associated miRNA can have pleiotropic effects on GDP [38, 46]. It represents as inversely correlations of a miRNA with traits of GDP and DT, potentially results in tradeoffs between DT and GDP. Based on our prediction, approximately ~ 14.2% of miRNAs (30 out of 211) can have potential negative pleiotropic effects on GDP. For instance, *OsmiRNA439(a-i)* negatively regulates *OsGDCH* (*LOC_Os10g37180*, PCC = -0.624) [26] while it positively regulates *OsPIN5b* (*LOC_Os08g41720*, PCC = 0.653) [30]. Moreover, *OsmiRNA439(a-i)* positively regulates *OsNAC5* (*LOC_Os11g08210,* PCC = 0.569) [19] and *OsALDH10A5* (*LOC_Os04g39020*, PCC = 0.582) [39], which act as positive regulators in DT. As a result, the upregulation of *OsmiRNA439(a-i)* under drought stress leads to inhibition of GDP and enhancement of DT by these DRGs.

Such a tradeoff could also be caused by a pair of mature miRNAs from the same pre-miRNA. These miRNAs commonly posttranscriptionally regulate different target genes [6], which may sometimes have opposite roles in regulating GDP and DT, as indicated by previous studies [37, 50] and by this study. This arrangement occurs at a frequency of 21.9% (nine pairs from type V out of 41 pairs in total) across the rice genome. We provide an example of *OsmiR1870-3p* and *OsmiR1870-5p* in this study. *OsmiR1870-3p* negatively regulates GDP while *OsmiR1870-5p* positively regulates DT via their different impacts on the rice transcriptome. Pre-*OsmiRNA408,* which forms two mature miRNAs (*OsmiRNA408-3p* and *OsmiRNA408-5p*), is another example*.* As reported by two independent studies, overexpression of *pre-OsmiRNA408* in rice decreases rice drought tolerance [37] while it positively regulates grain yield [50]. It has been reported that *OsmiRNA408-3p* positively regulates grain yield by suppressing *UCL8* (*LOC_Os03g50140*) [50]. We speculate that *OsmiRNA408-5p* negatively regulates drought tolerance by suppressing its predicted target gene *LOC_Os12g40890* (*OsIAA30*). Different roles of two mature miRNAs from the same pre-miRNA in DT and GDP raise a challenge for their utilization. We cannot simply manipulate the pre-miRNA, which is regularly applied in crop improvement [37, 50], to improve a given agronomic trait or obtain stress tolerance.

### Valuable candidates for the improvement of drought tolerance

While a miRNA can have systematic impacts on DT, it can sometimes have many pleiotropic effects [38, 46]. We should avoid unwanted pleiotropic effects on GDP by a DT gene when applying it in breeding strategies. In this study, we recommend some candidates of type II, which have low probabilities of unwanted pleiotropic effects on GDP. We also recommend four miRNAs of type III, which can be advantageous in both DT and GDP. Among all candidates, miRNA398a/b in *Medicago truncatula* [42] has been reported to be associated with DT. Three candidates, miRNA169g [55], miRNA398a [3], and miRNA5505 [49], have been recommended by other studies on rice. Given the considerable number of samples and the correlation-based analyses, we also identified many other potential candidates associated with DT. These miRNAs have great prospects in developing DT in rice.

## Conclusion

In this study, we detected 344 drought responsive miRNAs among six rice genotypes in total and described their temporal regulations along a progressive drought. These miRNAs can be divided into five categories based on their associations with drought tolerance and productivity. About 15% miRNAs possessed adverse impacts on rice drought tolerance and productivity, which limited their applications in breeding drought tolerant rice cultivars. Meanwhile, we also identified 21 drought responsive miRNAs that can have both advantages in drought tolerance and productivity once they were appropriately activated. The information of potential associations of miRNAs between rice drought-tolerance and productivity based on the correlation analysis can facilitate our utilization of miRNA in breeding.

## Methods

### Plant materials

Six rice genotypes were selected to investigate the genome-wide responses of miRNAs to long-term progressive drought, as well as their morphological and physiological performances. The materials were collected from International Rice Research Institute and conserved in the seed bank of Shanghai Agrobiological Gene Center (http://seed.sagc.org.cn/). The six rice genotypes have great differences in drought tolerance, which makes them a good system to study the association of miRNAs with drought tolerance. Based on our pre-evaluation and estimations in this study, genotype S3, S11, and S28 possess better drought tolerance, while genotype S9, S18, and S24 are susceptible to drought. Meanwhile, they are important breeding materials for water-saving and drought-resistant rice [31]. The knowledge of expression patterns of a drought-tolerant miRNA among the six genotypes can provide informative cues for us to utilize it by using the appropriate genotype in breeding. We made a minor adjustment in their dates of germination and transplanting to ensure they can meet the drought-stress before heading (Additional file 1: Table S14).

### Field experiment

The field experiment was conducted in a drought-resistance facility at Baihe Experimental Station, Shanghai, in 2014. The canopy of the facility was normally opened, and could be closed on rainy days to enable continuous drought. The depth of the soil layer in the drought-treated field was limited to 30 cm. With this design, the development of roots below this depth was equal between both cultivars and therefore the differences in drought-avoidance could be largely mitigated [32]. Moreover, planting rice in the experimental field rather than in pots led to more homogenous levels [32]. A similar field nearby was well-watered during the experiment as the control treatment (21.4% soil-water content). Plants for each genotype were planted in a plot of 10 rows × 10 hills at 18 cm intervals. Three replicates were set up for each genotype in the drought-treated and well-watered fields. The field arrangement was followed with a single factor randomized block design. The dates for germination and transplanting for the six genotypes were adjusted to make their heading during drought. We started the drought treatment on the 16th of July and continued the artificial drought for 38 days. We measured the soil water content at a depth of 30 cm in the drought-treated field every 3–5 days to monitor the progress of the drought. The drought-treated field was re-watered on the afternoon of the 22nd of August until the SWC decreased to ~ 10% (Additional file 2: Figure S10).

Physiological traits were measured using three replicates of leaf samples. Each replicate contained the three top leaves of the main tillers from each plot. A further three replicates of leaf samples were collected for miRNA and RNA sequencing. We harvested the leaf samples at six time points: the 24th of July (D1, tillering stage), the 29th of July (D2, booting stage), the 5th of August (D3, booting stage), the 11th of August (D4, heading stage), the 22nd of August (D5, heading stage), and the 23rd of August (R, recovery). All leaf samples were strictly collected between 13:00 and 14:00 for each time point and then put into liquid nitrogen immediately.

Osmolality were measured to reflect the osmotic adjustment of the leaf samples. We conducted the measurement of the osmolality via the Vapro™ vapor pressure osmometer (Wescor Model 5600). We measured the total antioxidant capacity (AOC), which reflects the capacity for ROS scavenging, using the total antioxidant capacity assay kit (product#A015, Nanjing Jiancheng Bioengineering Institute, Jiangsu, China). We measured the content of H_2_O_2_ and dead leaf ratio to reflect the physiological damage caused by drought stress. The content of H_2_O_2_ was measured by an H_2_O_2_ assay kits (product#A064, Nanjing Jiancheng Bioengineering Institute, Jiangsu, China). Measurements were taken from D1 to D5 during the drought period. The dead leaf ratio was estimated from five plants per plot on the 23rd of August. We also measured a number of important agronomic traits to reflect the growth, development, and reproduction of each genotype. Plant height and number of tillers were measured from five individual plants in each plot. The number of seeds, seed-setting ratio, biomass, and grain weight for each genotype were measured after harvest from eight plants per plot. Relative performances (the value of a trait in drought/that in CK) were calculated for these agronomic traits to reflect their DT. We applied independent *t*-tests to detect any significant differences in measured physiological and agronomic traits between the treatments at each time point.

### RNA extraction, library construction, and Illumina HiSeq sequencing

Total RNA was extracted from the leaf tissue using PureLink® Plant RNA Reagent (Life Technologies) according to the manufacturer’s instructions. Genomic DNA was removed using DNase I (Takara). Then, RNA quality was determined by a 2100 Bioanalyser (Agilent) and quantified using an ND-2000 (NanoDrop Technologies). Only high-quality RNA samples (OD260/280 = 1.8~2.2, OD260/230 ≥ 2.0, RIN ≥ 6.5, 28S:18S ≥ 1.0, > 10 μg) were used to construct a sequencing library. The concentration of RNA and the purity of the samples were estimated using a NanoDrop Spectrophotometer (Thermo Fisher Scientific) and Qubit Fluorometer (Thermo Fisher Scientific), respectively. An aliquot of the sample was also separated on an Agilent RNA Bioanalyzer chip to check for integrity. Libraries for sRNA sequencing were prepared with a TruSeq Small RNA Sample Preparation Guide (Illumina, USA) and sequenced on an Illumina NextSeq500 platform. Before library construction, three replicates of RNA samples from each genotype from one time point were mixed together. Total RNA libraries were constructed following the specifications in the TruSeq® RNA Sample Preparation v2 Guide (Illumina) and sequenced on an Illumina HiSeq 2500 platform. Three replicates of RNA samples for S3, S11, and S28 were mixed together to construct the libraries, while three replicates of RNA samples for S9, S18, and S24 were independently constructed from the libraries. High-throughput sequencing was conducted at Shanghai Majorbio Biopharm Technology Co., Ltd. (Shanghai, China). The raw data of miRNA (Additional file 1: Table S5) and mRNA (Additional file 1: Table S15) for each sample by high-throughput sequencing will be submitted to the NCBI Sequence Read Archive (SRA) under the number PRJNA609211 before publication.

### Data analysis

#### Identification and quantification of conserved miRNAs

The raw reads were trimmed and quality controlled by Fastx-Toolkit (http://hannonlab.cshl.edu/fastx_toolkit/). After the identical sequences were merged, all clean reads were separately aligned to the reference genome (Nipponbare, msu7.0) (http://rice.plantbiology.msu.edu/) using Bowtie 2 [23]. The reads that could be aligned to the reference genome were then compared to the database (http://www.mirbase.org/, Release 21, June 2014) to identify conserved miRNAs. We used miRDeep2 (https://www.mdc-berlin.de/content/mirdeep2-documentation) to count the clean reads and quantify the expression level of a detected miRNA by Transcripts per Million (TPM). A miRNA was used for further analysis if the TPM was > 0.10 in at least one sample. The expression data of the available miRNAs from each sample are provided in Additional file 4. The drought-responsive miRNA (DRMs) and recovery-related miRNAs (RRMs) for each genotype were determined using *p*-value< 0.05 and |log2FC| ≥ 1 during the drought period and at the recovery stage, respectively. We selected six DRMs to validate their expression in 10–15 randomly chosen samples by qPCR (Additional file 2: Figure S11). The primers for the miRNAs and the reference *U6* are listed in Additional file 1: Table S16.

#### Determination of differentially expressed genes between samples from CK and treated fields

We used SeqPrep to strip adaptors and/or merge paired reads with overlap into single reads (https://github.com/jstjohn/SeqPrep) and used Sickle to remove low-quality reads (https://github.com/najoshi/sickle). We then assembled the clean data using the software Cufflinks and mapped them to the reference genome (Nipponbare, msu7.0) and to mitochondrial and chloroplast genomes (http://rice.plantbiology.msu.edu/) via Tophat with no more than two base mismatches allowed in the alignment [41]. We determined the gene expression levels with the Fragment Per Kilobase of exon per Million fragments mapped (FPKM) method via the widely used software program Cuffdiff [41]. The gene expression quantified by FPKM can be well validated by qPCR in our previous study using the same samples (Additional file 2: Figure S12).

We determined differentially expressed genes (DEGs) between samples from drought and CK fields for each genotype at six time points. Since S9, S18, and S24 had three biological replicates, we determined their DEGs via a false discovery rate (FDR) < 0.05 and a logarithm two-fold change |log2FC| ≥ 1. Given the mixed nature of the cDNA libraries of S3, S11, and S28, we determined their DEGs with a *p*-value< 0.05 and |log2FC| ≥ 1. Moreover, as S9, S18, and S24 had three biological replicates, their FPKMs were averaged as a unit in further analysis. The expression data (FPKM) for all DEGs are provided in Additional file 5.

#### Studying impacts of miRNAs on the rice transcriptome, GDP, and DT by correlation analyses

To construct the regulatory network of the DRMs, correlation analyses (Pearson’s correlation coefficient, PCC) were applied to detect any significant correlations among DRMs based on their expressions. We predicted target genes for each miRNA by TargetFinder and psRobot to investigate the direct impacts of miRNAs on gene expression, particularly of genes known to be associated with GDP and/or DT (Additional file 2: Table S17). Detailed information can be found in the China Rice Data Center (http://www.ricedata.cn/gene/). Correlation analysis was further applied between the expression of DRMs and DEGs (from D1-D5 and R) to study the indirect impacts of miRNAs on the rice transcriptome. |PCC| > 0.6 and |PCC| > 0.4 was set as the threshold value to indicate a high and moderate correlation between a DRM and a DEG, respectively.

To predict the association of a DRM with GDP, correlation analyses were conducted between DRM expression and the measured agronomic and physiological traits among samples during drought (D1 to D5). We also conducted correlation analyses between the average fold change of a DRM during drought and four DT traits (relative no. of panicle, relative seed-setting ratio, relative biomass, and relative grain weight) to predict the association of the DRM with DT. To be more cautious, |PCC| > 0.6 was set as the threshold value to indicate the potential association of a DRM to a given trait. Based on the results of the correlation analysis, four types of DRMs were defined: (1) Type I, a DRM highly correlated with at least one GDP trait; (2) Type II, a DRM highly correlated with at least one DT trait; (3) Type III, a DRM positively or negatively correlated with both GDP and DT traits; and (4) Type IV, a DRM inversely correlated with at least one GDP trait and a DT trait.

We conducted enrichment analyses of Gene Ontology (GO) and KEGG pathways for each DRM based on its highly correlated DRGs. If a DRM had < 30 highly correlated DRGs, it was not included in the GO and KEGG enrichment analyses. The enriched GO biological processes (GOBPs) were further categorized into classifications by the web tool “GO Terms Classifications Counter” (http://www.animalgenome.org/cgi-bin/util/gotreei) [13]. We defined a preferential index (PI) to determine the preferential GO classifications for each type of DRM. PI is calculated as $\frac{\mathrm{Ng}/\mathrm{Nm}}{\mathrm{Ntg}/\mathrm{Ntm}}$, where N_g_ indicated no. of enriched GOBPs of a classification by one type of DRMs; N_m_ indicated no. of DRMs in this type; N_tg_ indicated no. of enriched GOBPs of a classification by total DRMs, and N_tm_ indicated no. of total DRMs.

#### Temporal gene regulation and relevant biological processes in tolerant and susceptible groups

Time course analyses on the regulation of miRNAs from D1 to D5 were conducted via hierarchical clustering (Euclidean method). Fold changes in the expression of each miRNA in the six genotypes from a single time point were averaged in this analysis. Based on this method, miRNAs could be divided into five major clusters based on the time course regulation of the miRNAs. We further defined five patterns of miRNA regulation at recovery (D5 to R): (A) upregulated at both D5 and recovery; (B) downregulated at both D5 and recovery; (C) upregulated at D5 while downregulated at recovery; (D) downregulated at D5 while upregulated at recovery; (E) regulation levels varied among genotypes. The preferences in GO classifications were also analyzed by the PI for RRMs in different patterns.

#### Validation of a predicted target gene of *osa-mi*R408-5p by qPCR

The fragment including the precursor of osa-miR408 was amplified from genomic DNA of upland rice cv. IRAT109 and placed to pCAMBIA1300-EGFP to control GFP expression. Then it was placed respectively to pCAMBIA1300-EGFP for fusion expression under the control of the CaMV35S promoter. And so, for the miR408 promoter driving GUS expression vector. All the constructs were transformed into the japonica rice cv. Zhonghua− 11 by the Agrobacterium-mediated (stain EHA105) transformation method42. Three transgenic lines (OX− 1~3) were used to test the expression of *osa-mR408-5p* and its target gene *LOC_Os12g40890* by qPCR. *U6* and *Actin* were used as the reference gene for miRNA and mRNA, respectively (Additional file 1: Table S16).

## Supplementary information

**Additional file 1: Table S1.** Agronomic traits measure among six rice genotypes in drought-treated and well-watered fields. * and ** indicate significant differences at *p* < 0.05 and *p* < 0.01 by independent *t* test between samples from drought and well-watered fields. NA is short for “not available”. **Table S2.** Content of H_2_O_2_ (mmol/g FW) measured among six rice genotypes in drought and well-watered fields. * indicates significant differences at *p* < 0.05 by independent *t* test between samples from drought and well-watered fields. **Table S3.** Osmotic potential measured among six rice genotypes in drought and well-watered fields. * and ** indicate significant differences at *p* < 0.05 and *p* < 0.01 by independent *t* test between samples from drought and well-watered fields. **Table S4.** Total anti-oxidant capacity (U/g FW) measured among six rice genotypes in drought and well-watered fields. * and ** indicate significant differences at *p* < 0.05 and *p* < 0.01 by independent *t* test between samples from drought and well-watered fields. **Table S5.** Basic information for sequenced samples. **Table S6.** Number of expressed, available, drought-responsive miRNAs (DRMs), and recovery-related miRNAs (RRMs). **Table S7.** Validation of expression patterns and correlation-based predictions by former functionally characterized miRNAs in rice. **Table S8.** Number of recovery-related miRNAs in five regulation patterns from timepoint D5 to recovery stage (R). **Table S9.** Number of recovery-related miRNAs (RRMs) in five regulation patterns from timepoint D5 to recovery stage (R). “Varied” indicates the regulation of a miRNA varies among genotypes. **Table S10.** Target genes predicted by TargetFinder and psRobot for drought-responsive miRNAs. GDP is short for growth, development, and reproduction. DT is short for drought tolerance. NA is short for not assigned. **Table S11.** Number of predicted target genes of differentially expressed miRNAs in drought responsive genes (DRGs) with different Pearson’s Correlation Coefficient (PCC) values. **Table S12.** Target genes predicted for miR408-3p and miR408-5p and their PPC values in correlations between miRNA and mRNA. **Table S13.** Pearson’s correlation coefficients (PCCs) of genes related to plant height (PH) and drought-tolerance (DT) with *OsmiR1870-3p* and *OsmiR1870-5p*. **Table S14.** Basic information for six rice genotypes. **Table S15.** Information for RNA-sequenced samples. **Table S16.** Primers of six miRNAs and the reference (U6) for qPCR. **Table S17.** List of GDP (growth, development, and reproduction)- and DT (drought-tolerance)-associated drought-responsive genes. These DT-associated genes have been functionally characterized. These GDP-associated genes are related to plant height, number of tillers, grain yield, and biomass by former studies.

**Additional file 2: Figure S1.** Distribution of the clean reads in small RNA libraries. **Figure S2.** Proportions of small RNAs in different length among genotypes (a), time points (b), and different treatments (c). **Figure S3.** A heatmap of the frequency of genotypes for drought-responsive miRNAs during drought (D1-D5). **Figure S4.** A heatmap of the frequency of time points (D1-D5) for drought-responsive miRNAs among six genotypes. **Figure S5.** Summary of drought-responsive miRNAs (DRMs) and recovery-related miRNAs (RRMs). **a**. Frequencies of a DRM among genotypes and time-points. **b**. Venn diagram of DRMs and RRMs. **Figure S6.** Expressions of *osa-mR408-5p* and its target gene *LOC_Os12g40890* quantified by qPCR in transgenic lines overexpressing *pre-miRNA408*. **Figure S7.** A heatmap describing the involvements of DRMs in biological processes based on the Gene Ontology enrichment using their highly correlated drought-responsive genes (|PCC > 0.6|). The blue (1) color indicates significant enrichment (FDR < 0.05), while the grey (0) color indicates no significant enrichment (*p* > 0.05). **Figure S8.** A heatmap describing the involvements of DRMs in metabolic pathways based on the KEGG enrichment using their highly correlated drought-responsive genes (|PCC > 0.6|). The blue (1) color indicates the significant enrichment (*p* < 0.05), while the grey (0) color indicates no significant enrichment (*p* > 0.05). **Figure S9.** Impacts of *OsmiR1870-3p* and *OsmiR1870-5p* on rice transcriptome. a. Venn diagram of positively (PCC > 0.4) and negatively (PCC < -0.4) correlated drought-responsive genes (DRGs) for *OsmiR1870-3p* and *OsmiR1870-5p.* b. GO enrichment by correlated DRGs of *OsmiR1870-3p*. c. GO enrichment by correlated DRGs of *OsmiR1870-5p*. PCC, Pearson’s correlation coefficient. GO terms in red, orange, and yellow indicate *p* < 0.001. *p* < 0.01, and *p* < 0.05 in the enrichment analyses, respectively. **Figure S10.** Soil-water content measured during drought. **Figure S11.** Fold changes (drought/ well-watered) of six drought-responsive miRNAs quantified by high-throughput sequencing is well validated by qPCR. **Figure S12.** Correlations between expressions of six drought responsive genes quantified by RNA-seq and qPCR. The data can be also found in the reference Ma et al. [29].

**Additional file 3.** Matrix of Pearson’s correlation coefficients (PCCs) between drought-responsive miRNAs and drought-responsive genes.

**Additional file 4.** Expressions (by TPM) of detected miRNAs.

**Additional file 5.** Expressions (by FPKM) of drought-responsive genes.

## Data Availability

All data supporting the conclusions of this article are provided within the article and its Additional files 1, 2, 3, 4 and 5. Raw data of sequenced sample will be submitted to the NCBI Sequence Read Archive (SRA) under the number PRJNA609211 before publication.

## Abbreviations

DRM: Drought-responsive miRNA
DT: Drought tolerance
GDP: Growth, development, and reproduction
GO: Gene Ontology
GOBP: Biological processes in Gene Ontology
KEGG: Kyoto Encyclopedia of Genes and Genomes
PCC: Pearson correlation coefficient
PI: Preferential index
RRM: Recovery-related miRNA
