# Supplementary material for: Temporal responses of conserved miRNAs to drought and their associations with drought tolerance and productivity in rice
Source: BMC Genomics. 2020 Mar 14;21:232. doi: 10.1186/s12864-020-6646-5 (PMC7071783; doi:10.1186/s12864-020-6646-5)
Supplement: Supplementary file 2 — Additional file 2: Figure S1. Distribution of the clean reads in small RNA libraries. Figure S2. Proportions of small RNAs in different length among genotypes (a), time points (b), and different treatments (c). Figure S3. A heatmap of the frequency of genotypes for drought-responsive miRNAs during drought (D1-D5). Figure S4. A heatmap of the frequency of time points (D1-D5) for drought-responsive miRNAs among six genotypes. Figure S5. Summary of drought-responsive miRNAs (DRMs) and recovery-related miRNAs (RRMs). a. Frequencies of a DRM among genotypes and time-points. b. Venn diagram of DRMs and RRMs. Figure S6. Expressions of osa-mR408-5p and its target gene LOC_Os12g40890 quantified by qPCR in transgenic lines overexpressing pre-miRNA408. Figure S7. A heatmap describing the involvements of DRMs in biological processes based on the Gene Ontology enrichment using their highly correlated drought-responsive genes (|PCC > 0.6|). The blue (1) color indicates significant enrichment (FDR < 0.05), while the grey (0) color indicates no significant enrichment (p > 0.05). Figure S8. A heatmap describing the involvements of DRMs in metabolic pathways based on the KEGG enrichment using their highly correlated drought-responsive genes (|PCC > 0.6|). The blue (1) color indicates the significant enrichment (p < 0.05), while the grey (0) color indicates no significant enrichment (p > 0.05). Figure S9. Impacts of OsmiR1870-3p and OsmiR1870-5p on rice transcriptome. a. Venn diagram of positively (PCC > 0.4) and negatively (PCC < -0.4) correlated drought-responsive genes (DRGs) for OsmiR1870-3p and OsmiR1870-5p. b. GO enrichment by correlated DRGs of OsmiR1870-3p. c. GO enrichment by correlated DRGs of OsmiR1870-5p. PCC, Pearson’s correlation coefficient. GO terms in red, orange, and yellow indicate p < 0.001. p < 0.01, and p < 0.05 in the enrichment analyses, respectively. Figure S10. Soil-water content measured during drought. Figure S11. Fold changes (drought/ well-watered) of six [file 12864_2020_6646_MOESM2_ESM.pdf]

Supplementary Figure Legends

Figure S1. Distribution of the clean reads in small RNA libraries

Figure S2. Proportions of small RNAs in different length among genotypes (a), time points (b), and different treatments (c).

Figure S3. A heatmap of the frequency of genotypes for drought-responsive miRNAs during drought (D1-D5).

Figure S4. A heatmap of the frequency of time points (D1-D5) for drought-responsive miRNAs among six genotypes.

Figure S5. Summary of drought-responsive miRNAs (DRMs) and recovery-related miRNAs (RRMs). **a**. Frequencies of a DRM among genotypes and time-points. **b**. Venn diagram of DRMs and RRMs.

Figure S6. Expressions of *osa-mR408-5p* and its target gene *LOC_Os12g40890* quantified by qPCR in transgenic lines (OX-1~3) overexpressing *pre-miRNA408*. *** indicates significance at p<0.001 by independent *t* test.

Figure S7. A heatmap describing the involvements of DRMs in biological processes based on the Gene Ontology enrichment using their highly correlated drought-responsive genes (|PCC>0.6|). The blue (1) color indicates significant enrichment (FDR <0.05), while the grey (0) color indicates no significant enrichment (*p*>0.05).

Figure S8. A heatmap describing the involvements of DRMs in metabolic pathways based on the KEGG enrichment using their highly correlated drought-responsive genes (|PCC>0.6|). The blue (1) color indicates the significant enrichment (*p* <0.05), while the grey (0) color indicates no significant enrichment (*p*>0.05).

Figure S9. Impacts of *OsmiR1870-3p* and *OsmiR1870-5p* on rice transcriptome. a. Venn diagram of positively (PCC>0.4) and negatively (PCC<-0.4) correlated drought-responsive genes (DRGs) for *OsmiR1870-3p* and *OsmiR1870-5p.* b. GO enrichment by correlated DRGs of *OsmiR1870-3p*. c. GO enrichment by correlated DRGs of *OsmiR1870-5p*. PCC, Pearson's correlation coefficient. GO terms in red, orange, and yellow indicate p<0.001. p<0.01, and p<0.05 in the enrichment analyses, respectively.

Figure S10. Soil-water content measured during drought.

Figure S11. Fold changes (drought/ well-watered) of six drought-responsive miRNAs quantified by high-throughput sequencing is well validated by qPCR.

Figure S12. Correlations between expressions of six drought responsive genes quantified by RNA-seq and qPCR. The data can be also found in the reference Ma et al. (2016).

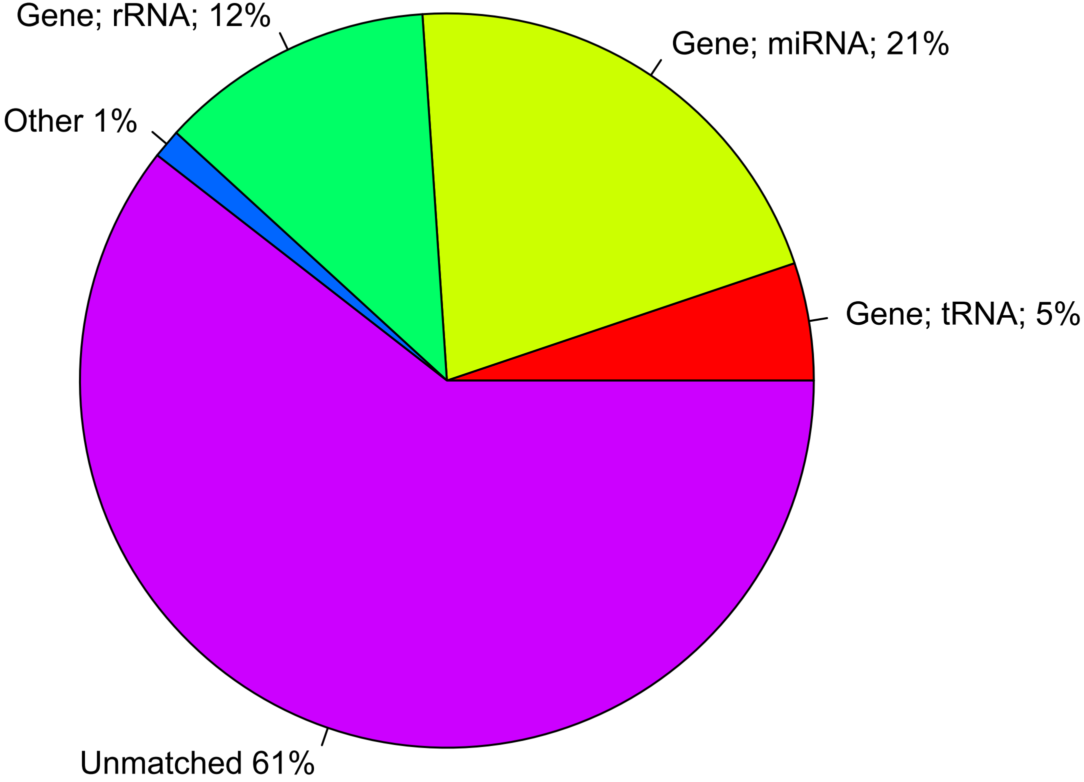
Gene; rRNA; 12%
Gene; miRNA; 21%
Other 1%
Gene; tRNA; 5%
Unmatched 61%

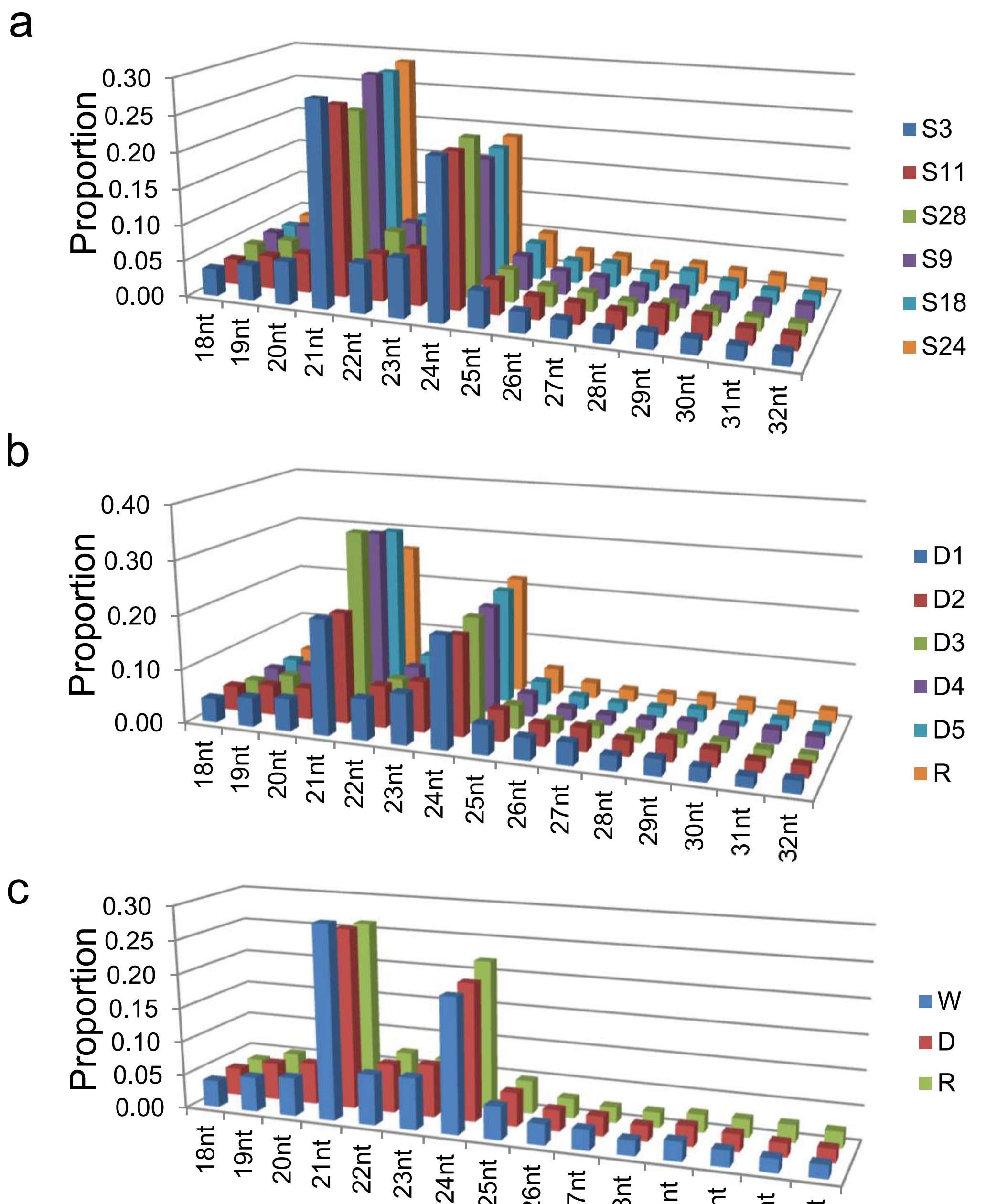
a
Proportion
0.30
0.25
0.20
0.15
0.10
0.05
0.00
18nt
19nt
20nt
21nt
22nt
23nt
24nt
25nt
26nt
27nt
28nt
29nt
30nt
31nt
32nt
S3
S11
S28
S9
S18
S24
b
Proportion
0.40
0.30
0.20
0.10
0.00
D1
D2
D3
D4
D5
R
c
Proportion
0.30
0.25
0.20
0.15
0.10
0.05
0.00
W
D
R

Fig. S2

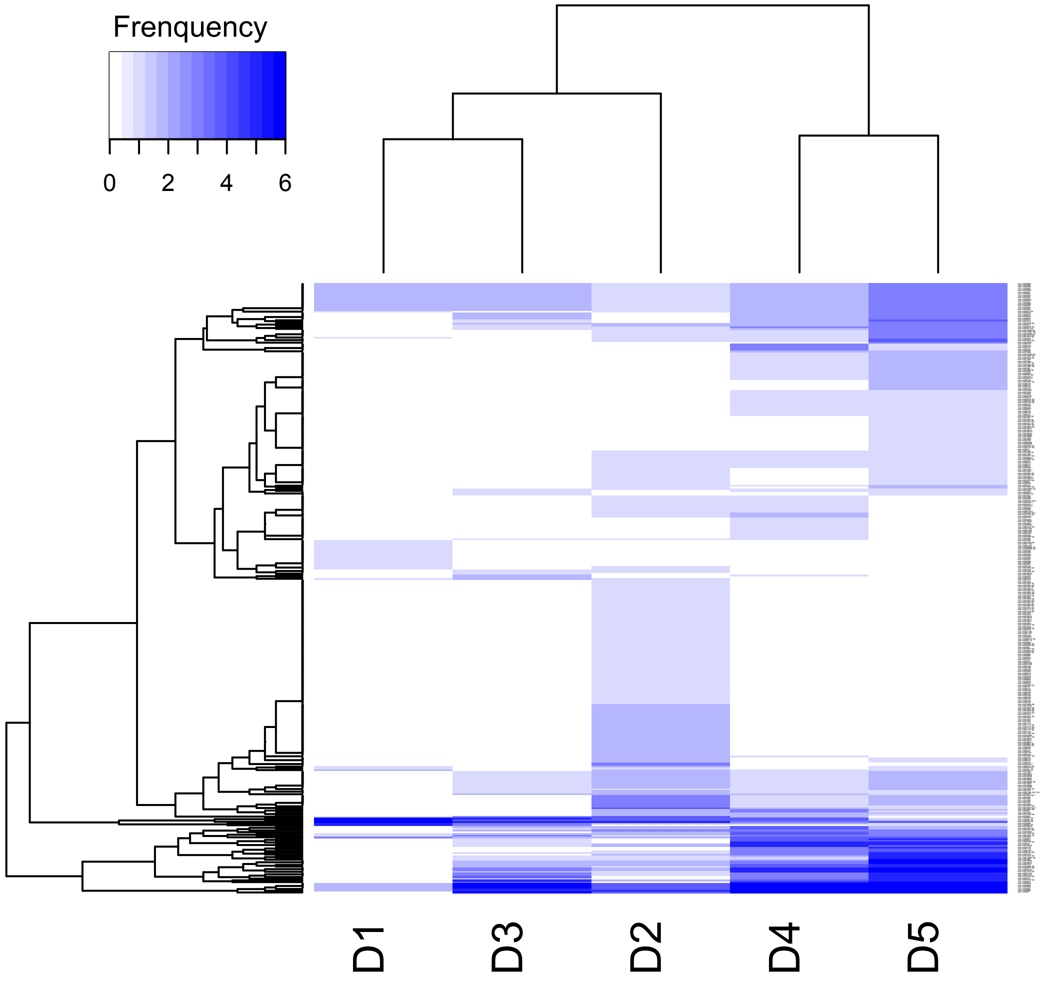

Frenquency
0
2
4
6
D1
D3
D2
D4
D5

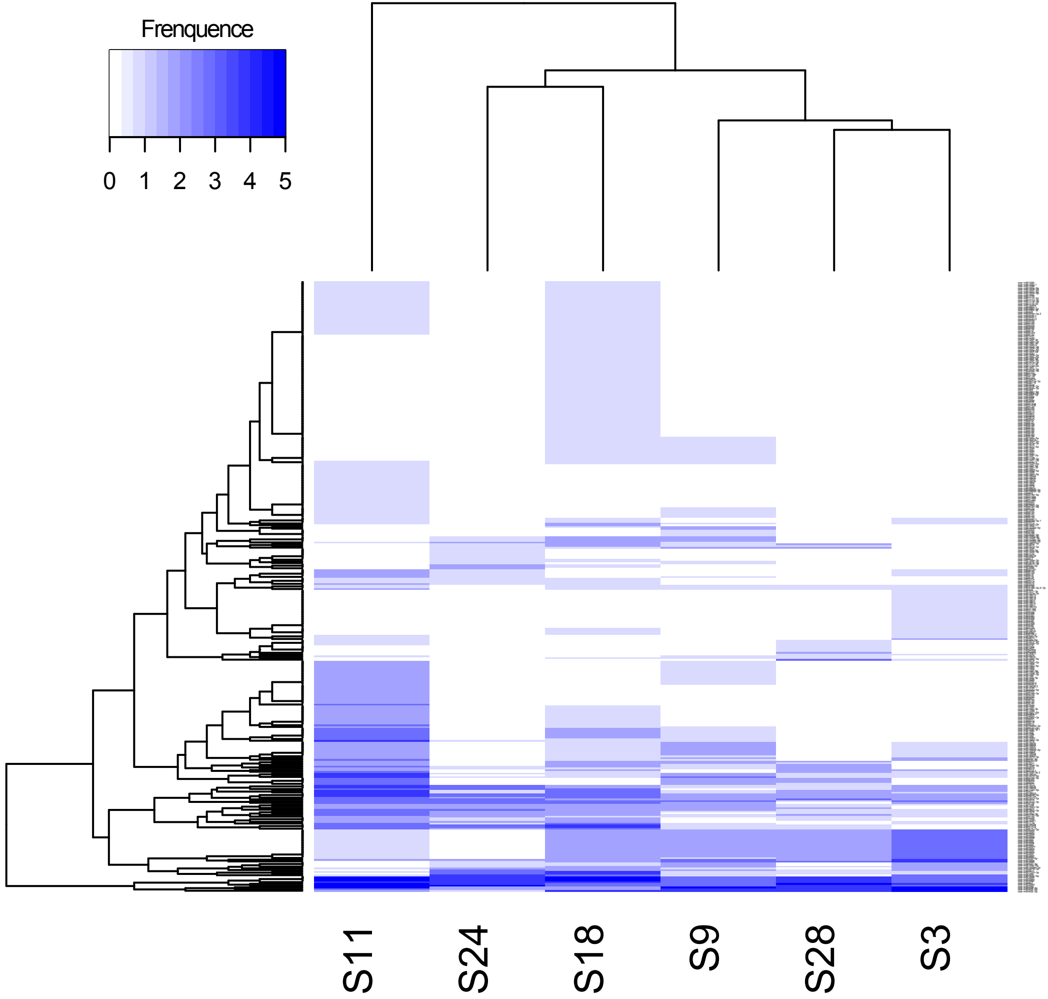
Frenquence
0
1
2
3
4
5
S11
S24
S18
S9
S28
S3

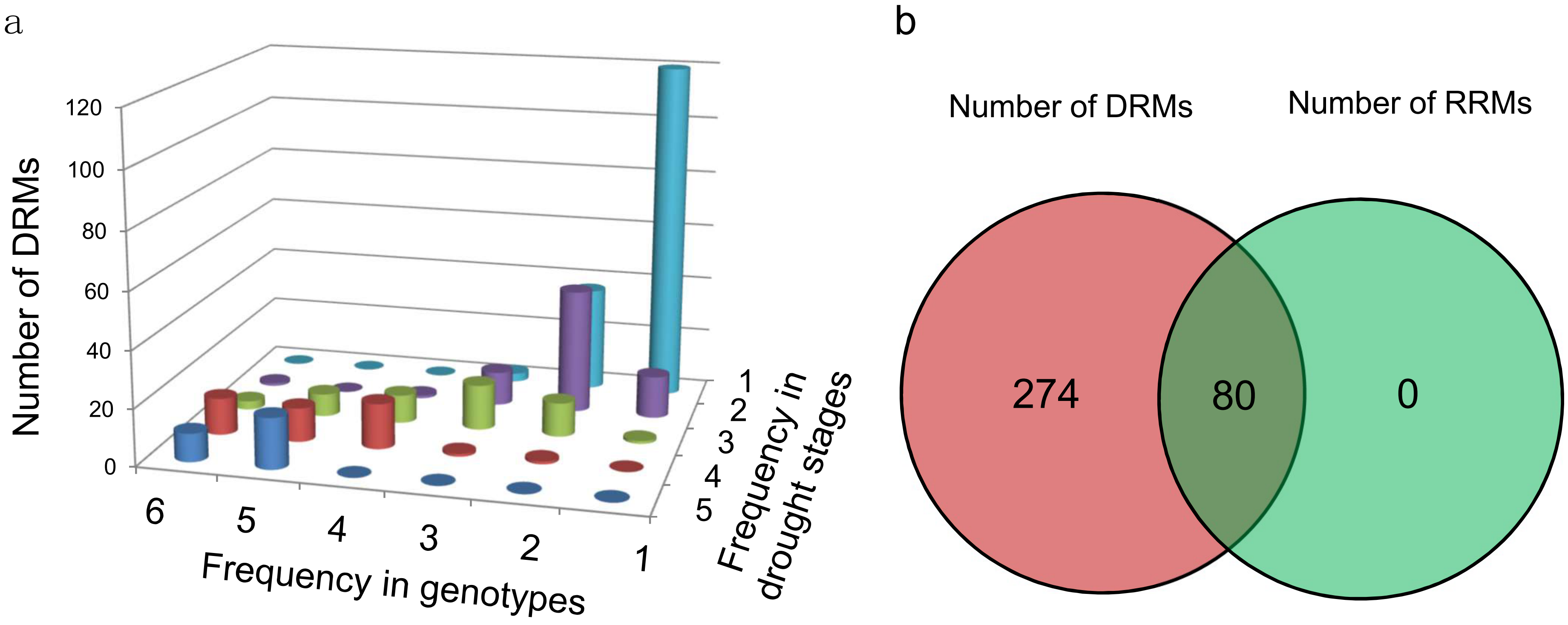
a
Number of DRMs
120
100
80
60
40
20
0
6
5
4
3
2
1
Frequency in genotypes
1
2
3
4
5
Frequency in drought stages
b
Number of DRMs
Number of RRMs
274
80
0

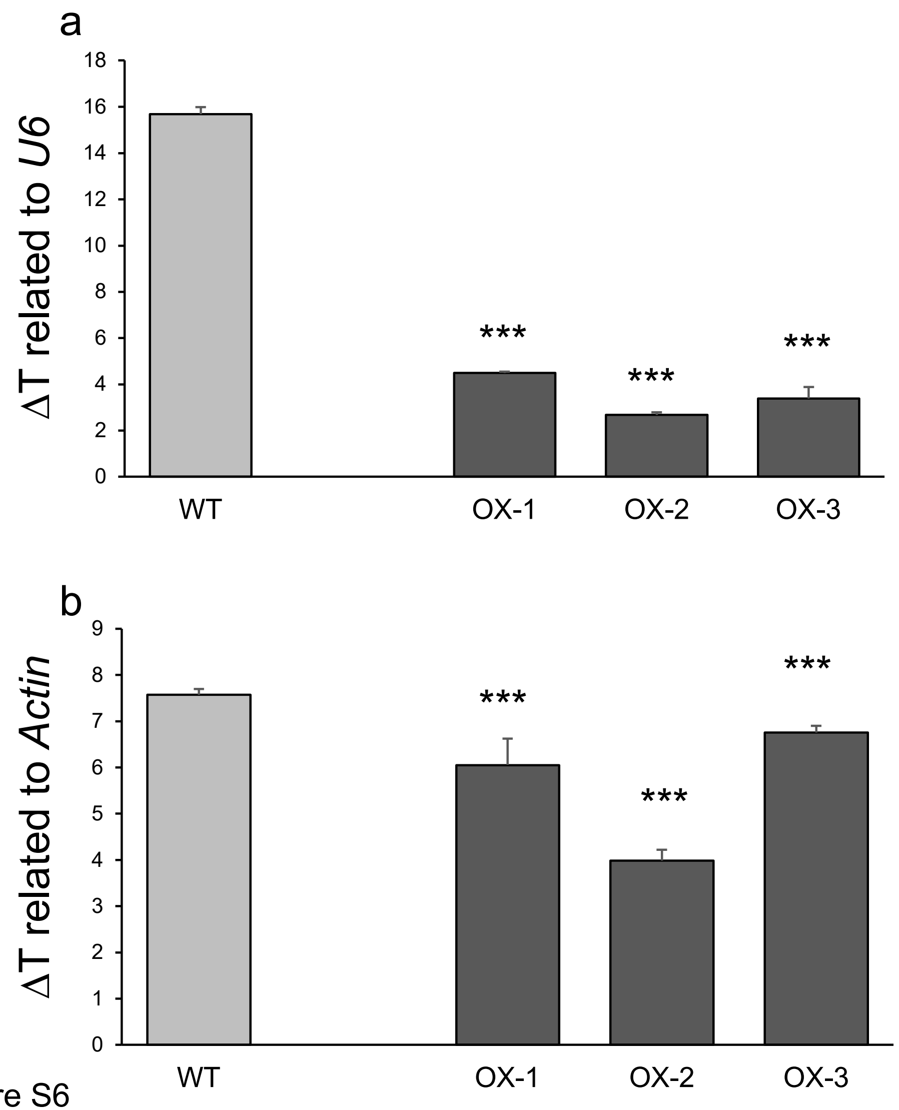
a
ΔT related to U6
18
16
14
12
10
8
6
4
2
0
***
***
***
WT
OX-1
OX-2
OX-3
b
ΔT related to Actin
9
8
7
6
5
4
3
2
1
0
***
***
***
WT
OX-1
OX-2
OX-3
Figure S6

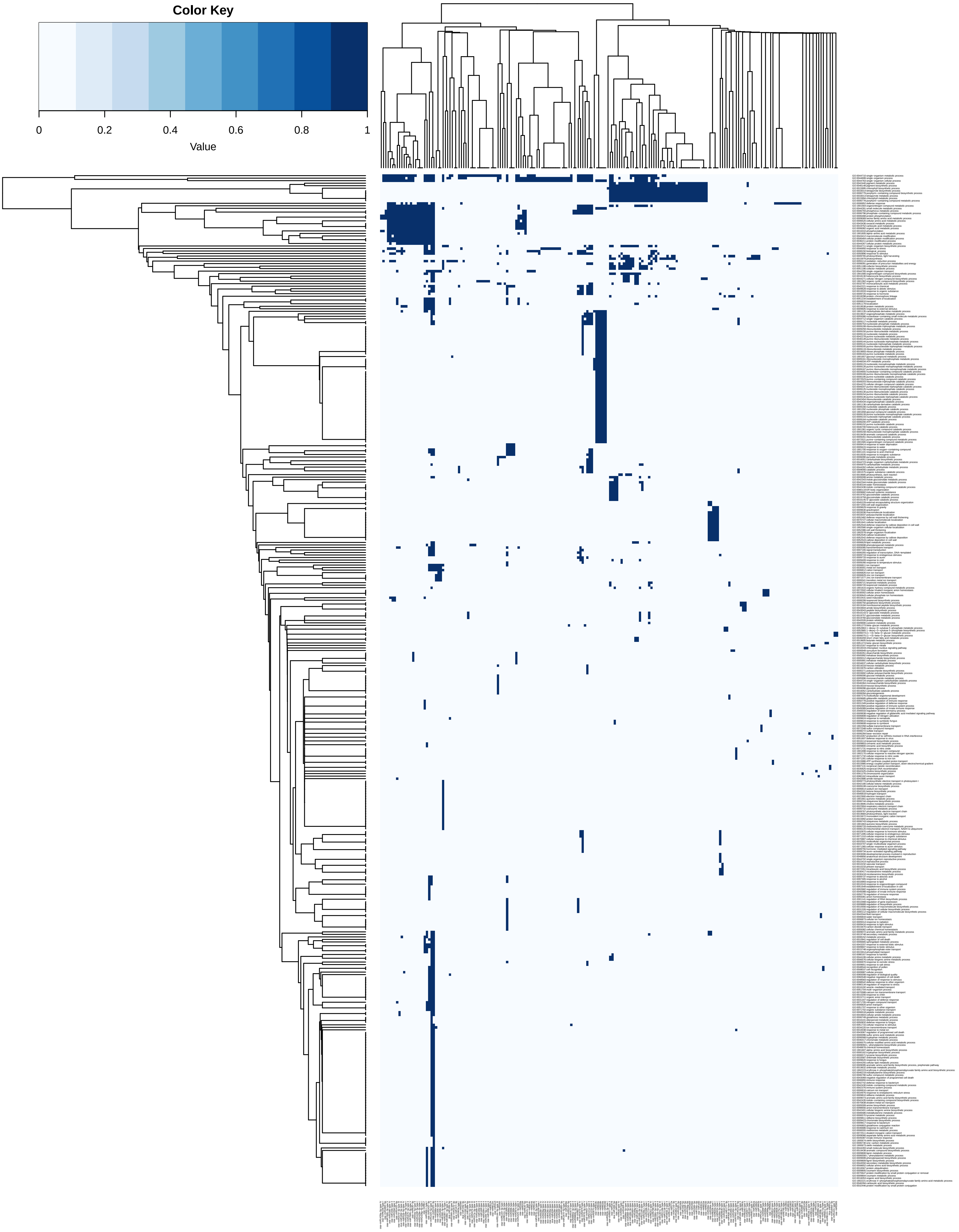
Color Key
0
0.2
0.4
0.6
0.8
1
Value

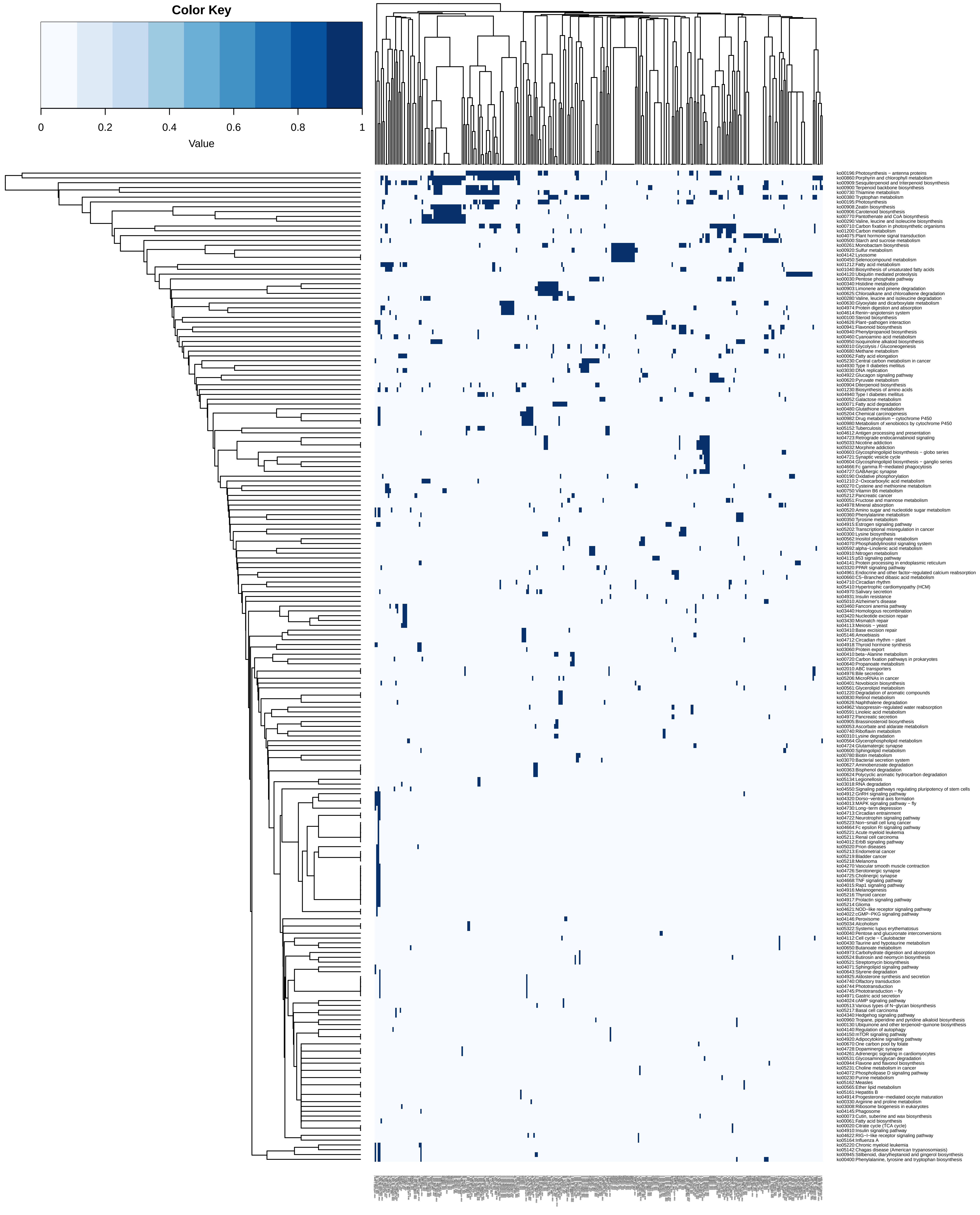
Color Key
0
0.2
0.4
0.6
0.8
1
Value
ko00196:Photosynthesis − antenna proteins
ko00860:Porphyrin and chlorophyll metabolism
ko00909:Sesquiterpenoid and triterpenoid biosynthesis
ko00900:Terpenoid backbone biosynthesis
ko00730:Thiamine metabolism
ko00380:Tryptophan metabolism
ko00195:Photosynthesis
ko00908:Zeatin biosynthesis
ko00906:Carotenoid biosynthesis
ko00770:Pantothenate and CoA biosynthesis
ko00290:Valine, leucine and isoleucine biosynthesis
ko00710:Carbon fixation in photosynthetic organisms
ko01200:Carbon metabolism
ko04075:Plant hormone signal transduction
ko00500:Starch and sucrose metabolism
ko00261:Monobactam biosynthesis
ko00920:Sulfur metabolism
ko04142:Lysosome
ko00450:Selenocompound metabolism
ko01212:Fatty acid metabolism
ko01040:Biosynthesis of unsaturated fatty acids
ko04120:Ubiquitin mediated proteolysis
ko00030:Pentose phosphate pathway
ko00340:Histidine metabolism
ko00903:Limonene and pinene degradation
ko00625:Chloroalkane and chloroalkene degradation
ko00280:Valine, leucine and isoleucine degradation
ko00630:Glyoxylate and dicarboxylate metabolism
ko04974:Protein digestion and absorption
ko04614:Renin−angiotensin system
ko00100:Steroid biosynthesis
ko04626:Plant−pathogen interaction
ko00941:Flavonoid biosynthesis
ko00940:Phenylpropanoid biosynthesis
ko00460:Cyanoamino acid metabolism
ko00950:Isoquinoline alkaloid biosynthesis
ko00010:Glycolysis / Gluconeogenesis
ko00680:Methane metabolism
ko00062:Fatty acid elongation
ko05230:Central carbon metabolism in cancer
ko04930:Type II diabetes mellitus
ko03030:DNA replication
ko04922:Glucagon signaling pathway
ko00620:Pyruvate metabolism
ko00904:Diterpenoid biosynthesis
ko01230:Biosynthesis of amino acids
ko04940:Type I diabetes mellitus
ko00052:Galactose metabolism
ko00071:Fatty acid degradation
ko00480:Glutathione metabolism
ko05204:Chemical carcinogenesis
ko00982:Drug metabolism − cytochrome P450
ko00980:Metabolism of xenobiotics by cytochrome P450
ko05152:Tuberculosis
ko04612:Antigen processing and presentation
ko04723:Retrograde endocannabinoid signaling
ko05033:Nicotine addiction
ko05032:Morphine addiction
ko00603:Glycosphingolipid biosynthesis − globo series
ko04721:Synaptic vesicle cycle
ko00604:Glycosphingolipid biosynthesis − ganglio series
ko04666:Fc gamma R−mediated phagocytosis
ko04727:GABAergic synapse
ko00190:Oxidative phosphorylation
ko01210:2−Oxocarboxylic acid metabolism
ko00270:Cysteine and methionine metabolism
ko00750:Vitamin B6 metabolism
ko05212:Pancreatic cancer
ko00051:Fructose and mannose metabolism
ko04978:Mineral absorption
ko00520:Amino sugar and nucleotide sugar metabolism
ko00360:Phenylalanine metabolism
ko00350:Tyrosine metabolism
ko04915:Estrogen signaling pathway
ko05202:Transcriptional misregulation in cancer
ko00300:Lysine biosynthesis
ko00562:Inositol phosphate metabolism
ko04070:Phosphatidylinositol signaling system
ko00592:alpha−Linolenic acid metabolism
ko00910:Nitrogen metabolism
ko04115:p53 signaling pathway
ko04141:Protein processing in endoplasmic reticulum
ko03320:PPAR signaling pathway
ko04961:Endocrine and other factor−regulated calcium reabsorption
ko00660:C5−Branched dibasic acid metabolism
ko04710:Circadian rhythm
ko05410:Hypertrophic cardiomyopathy (HCM)
ko04970:Salivary secretion
ko04931:Insulin resistance
ko05010:Alzheimer's disease
ko03460:Fanconi anemia pathway
ko03440:Homologous recombination
ko03420:Nucleotide excision repair
ko03430:Mismatch repair
ko04113:Meiosis − yeast
ko03410:Base excision repair
ko05146:Amoebiasis
ko04712:Circadian rhythm − plant
ko04918:Thyroid hormone synthesis
ko03060:Protein export
ko00410:beta−Alanine metabolism
ko00720:Carbon fixation pathways in prokaryotes
ko00640:Propanoate metabolism
ko02010:ABC transporters
ko04976:Bile secretion
ko05206:MicroRNAs in cancer
ko00401:Novobiocin biosynthesis
ko00561:Glycerolipid metabolism
ko01220:Degradation of aromatic compounds
ko00830:Retinol metabolism
ko00626:Naphthalene degradation
ko04962:Vasopressin−regulated water reabsorption
ko00591:Linoleic acid metabolism
ko04972:Pancreatic secretion
ko00905:Brassinosteroid biosynthesis
ko00053:Ascorbate and aldarate metabolism
ko00740:Riboflavin metabolism
ko00310:Lysine degradation
ko00564:Glycerophospholipid metabolism
ko04724:Glutamatergic synapse
ko00600:Sphingolipid metabolism
ko00780:Biotin metabolism
ko03070:Bacterial secretion system
ko00627:Aminobenzoate degradation
ko00363:Bisphenol degradation
ko00624:Polycyclic aromatic hydrocarbon degradation
ko05134:Legionellosis
ko03018:RNA degradation
ko04550:Signaling pathways regulating pluripotency of stem cells
ko04912:GnRH signaling pathway
ko04320:Dorso−ventral axis formation
ko04013:MAPK signaling pathway − fly
ko04730:Long−term depression
ko04713:Circadian entrainment
ko04722:Neurotrophin signaling pathway
ko05223:Non−small cell lung cancer
ko04664:Fc epsilon RI signaling pathway
ko05221:Acute myeloid leukemia
ko05211:Renal cell carcinoma
ko04012:ErbB signaling pathway
ko05020:Prion diseases
ko05213:Endometrial cancer
ko05219:Bladder cancer
ko05218:Melanoma
ko04270:Vascular smooth muscle contraction
ko04726:Serotonergic synapse
ko04725:Cholinergic synapse
ko04668:TNF signaling pathway
ko04015:Rap1 signaling pathway
ko04916:Melanogenesis
ko05216:Thyroid cancer
ko04917:Prolactin signaling pathway
ko05214:Glioma
ko04621:NOD−like receptor signaling pathway
ko04022:cGMP−PKG signaling pathway
ko04146:Peroxisome
ko05034:Alcoholism
ko05322:Systemic lupus erythematosus
ko00040:Pentose and glucuronate interconversions
ko04112:Cell cycle − Caulobacter
ko00430:Taurine and hypotaurine metabolism
ko00650:Butanoate metabolism
ko04973:Carbohydrate digestion and absorption
ko00524:Butirosin and neomycin biosynthesis
ko00521:Streptomycin biosynthesis
ko04071:Sphingolipid signaling pathway
ko00643:Styrene degradation
ko04925:Aldosterone synthesis and secretion
ko04740:Olfactory transduction
ko04744:Phototransduction
ko04745:Phototransduction − fly
ko04971:Gastric acid secretion
ko04024:cAMP signaling pathway
ko00513:Various types of N−glycan biosynthesis
ko05217:Basal cell carcinoma
ko04340:Hedgehog signaling pathway
ko00960:Tropane, piperidine and pyridine alkaloid biosynthesis
ko00130:Ubiquinone and other terpenoid−quinone biosynthesis
ko04140:Regulation of autophagy
ko04150:mTOR signaling pathway
ko04920:Adipocytokine signaling pathway
ko00670:One carbon pool by folate
ko04728:Dopaminergic synapse
ko04261:Adrenergic signaling in cardiomyocytes
ko00531:Glycosaminoglycan degradation
ko00944:Flavone and flavonol biosynthesis
ko05231:Choline metabolism in cancer
ko04072:Phospholipase D signaling pathway
ko00230:Purine metabolism
ko05162:Measles
ko00565:Ether lipid metabolism
ko05161:Hepatitis B
ko04914:Progesterone−mediated oocyte maturation
ko00330:Arginine and proline metabolism
ko03008:Ribosome biogenesis in eukaryotes
ko04145:Phagosome
ko00073:Cutin, suberine and wax biosynthesis
ko00061:Fatty acid biosynthesis
ko00020:Citrate cycle (TCA cycle)
ko04910:Insulin signaling pathway
ko04622:RIG−I−like receptor signaling pathway
ko05164:Influenza A
ko05220:Chronic myeloid leukemia
ko05142:Chagas disease (American trypanosomiasis)
ko00945:Stilbenoid, diarylheptanoid and gingerol biosynthesis
ko00400:Phenylalanine, tyrosine and tryptophan biosynthesis

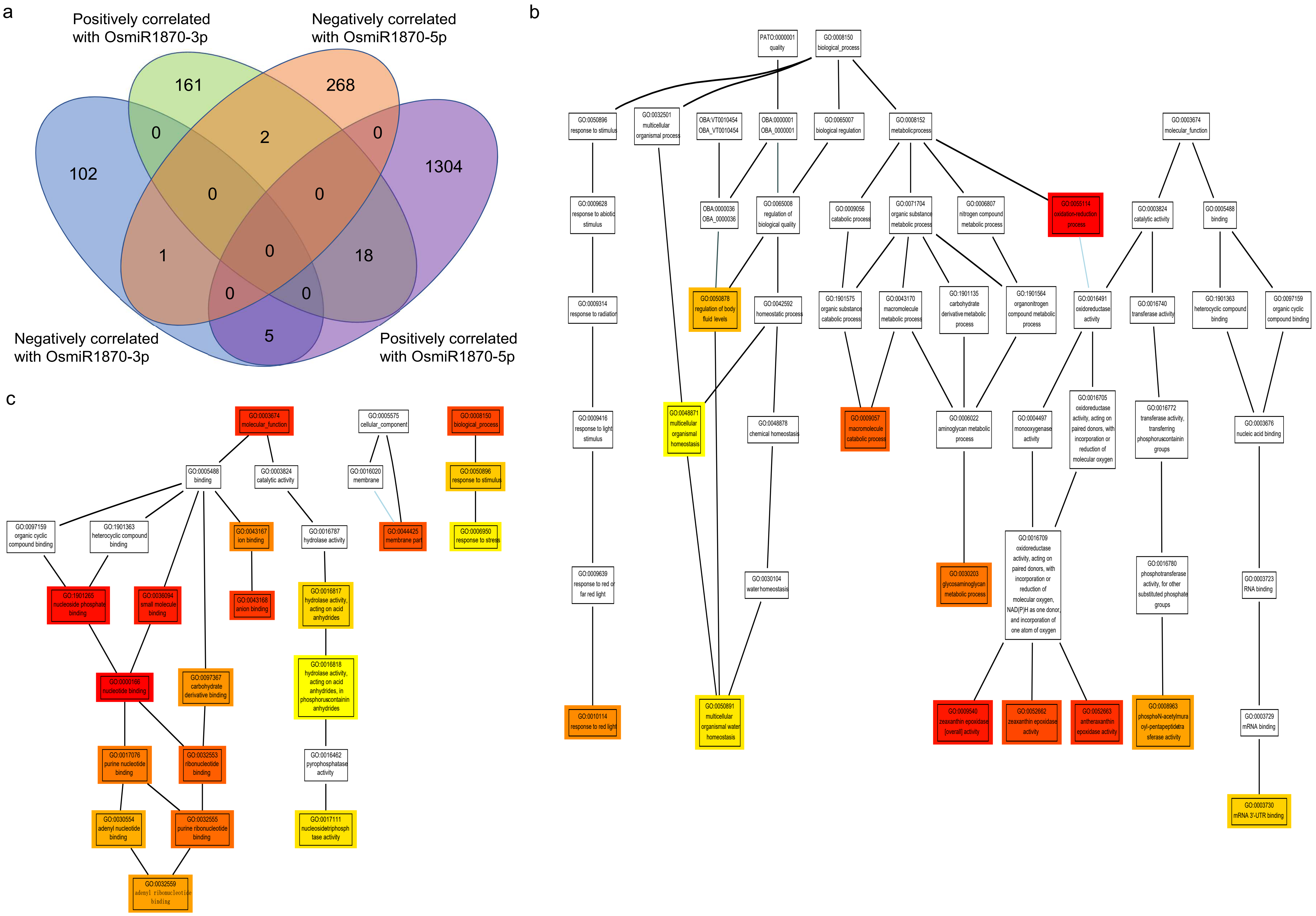
a
Positively correlated with OsmiR1870-3p
Negatively correlated with OsmiR1870-5p
Negatively correlated with OsmiR1870-3p
Positively correlated with OsmiR1870-5p
161
268
102
1304
0
2
0
0
0
1
0
18
0
0
5
b
PATO:0000001 quality
GO:0008150 biological_process
GO:0050896 response to stimulus
GO:0032501 multicellular organismal process
OBA:VT0010454 OBA_VT0010454
OBA:0000001 OBA_0000001
GO:0065007 biological regulation
GO:0008152 metabolic process
GO:0003674 molecular_function
GO:0009628 response to abiotic stimulus
OBA:0000036 OBA_0000036
GO:0065008 regulation of biological quality
GO:0009056 catabolic process
GO:0071704 organic substance metabolic process
GO:0006807 nitrogen compound metabolic process
GO:0055114 oxidation-reduction process
GO:0003824 catalytic activity
GO:0005488 binding
GO:0009314 response to radiation
GO:0050878 regulation of body fluid levels
GO:0042592 homeostatic process
GO:1901575 organic substance catabolic process
GO:0043170 macromolecule metabolic process
GO:1901135 carbohydrate derivative metabolic process
GO:1901564 organonitrogen compound metabolic process
GO:0016491 oxidoreductase activity
GO:0016740 transferase activity
GO:1901363 heterocyclic compound binding
GO:0097159 organic cyclic compound binding
GO:0009416 response to light stimulus
GO:0048871 multicellular organismal homeostasis
GO:0048878 chemical homeostasis
GO:0009057 macromolecule catabolic process
GO:0006022 aminoglycan metabolic process
GO:0004497 monooxygenase activity
GO:0016705 oxidoreductase activity, acting on paired donors, with incorporation or reduction of molecular oxygen
GO:0016772 transferase activity, transferring phosphorus-containing groups
GO:0003676 nucleic acid binding
GO:0009639 response to red or far red light
GO:0030104 water homeostasis
GO:0030203 glycosaminoglycan metabolic process
GO:0016709 oxidoreductase activity, acting on paired donors, with incorporation or reduction of molecular oxygen, NAD(P)H as one donor, and incorporation of one atom of oxygen
GO:0016780 phosphotransferase activity, for other substituted phosphate groups
GO:0003723 RNA binding
GO:0010114 response to red light
GO:0050891 multicellular organismal water homeostasis
GO:0009540 zeaxanthin epoxidase [overall] activity
GO:0052662 zeaxanthin epoxidase activity
GO:0052663 antheraxanthin epoxidase activity
GO:0008963 phospho-N-acetylmuramoyl-pentapeptide-transferase activity
GO:0003729 mRNA binding
GO:0003730 mRNA 3'-UTR binding
c
GO:0003674 molecular_function
GO:0005575 cellular_component
GO:0008150 biological_process
GO:0005488 binding
GO:0003824 catalytic activity
GO:0016020 membrane
GO:0050896 response to stimulus
GO:0097159 organic cyclic compound binding
GO:1901363 heterocyclic compound binding
GO:0043167 ion binding
GO:0016787 hydrolase activity
GO:0044425 membrane part
GO:0006950 response to stress
GO:1901265 nucleoside phosphate binding
GO:0036094 small molecule binding
GO:0043168 anion binding
GO:0016817 hydrolase activity, acting on acid anhydrides
GO:0016818 hydrolase activity, acting on acid anhydrides, in phosphorus-containing anhydrides
GO:0000166 nucleotide binding
GO:0097367 carbohydrate derivative binding
GO:0017076 purine nucleotide binding
GO:0032553 ribonucleotide binding
GO:0016462 pyrophosphatase activity
GO:0030554 adenyl nucleotide binding
GO:0032555 purine ribonucleotide binding
GO:0017111 nucleoside-triphosphatase activity
GO:0032559 adenyl ribonucleotide binding

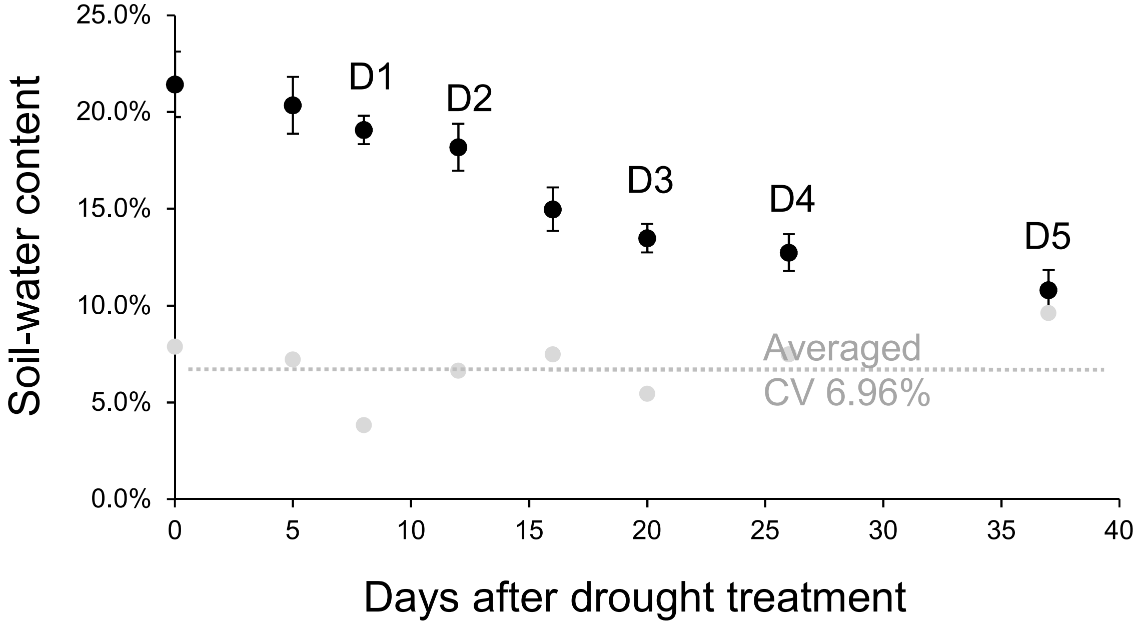

25.0%
20.0%
15.0%
10.0%
5.0%
0.0%
Soil-water content
D1
D2
D3
D4
D5
Averaged
CV 6.96%
0
5
10
15
20
25
30
35
40
Days after drought treatment

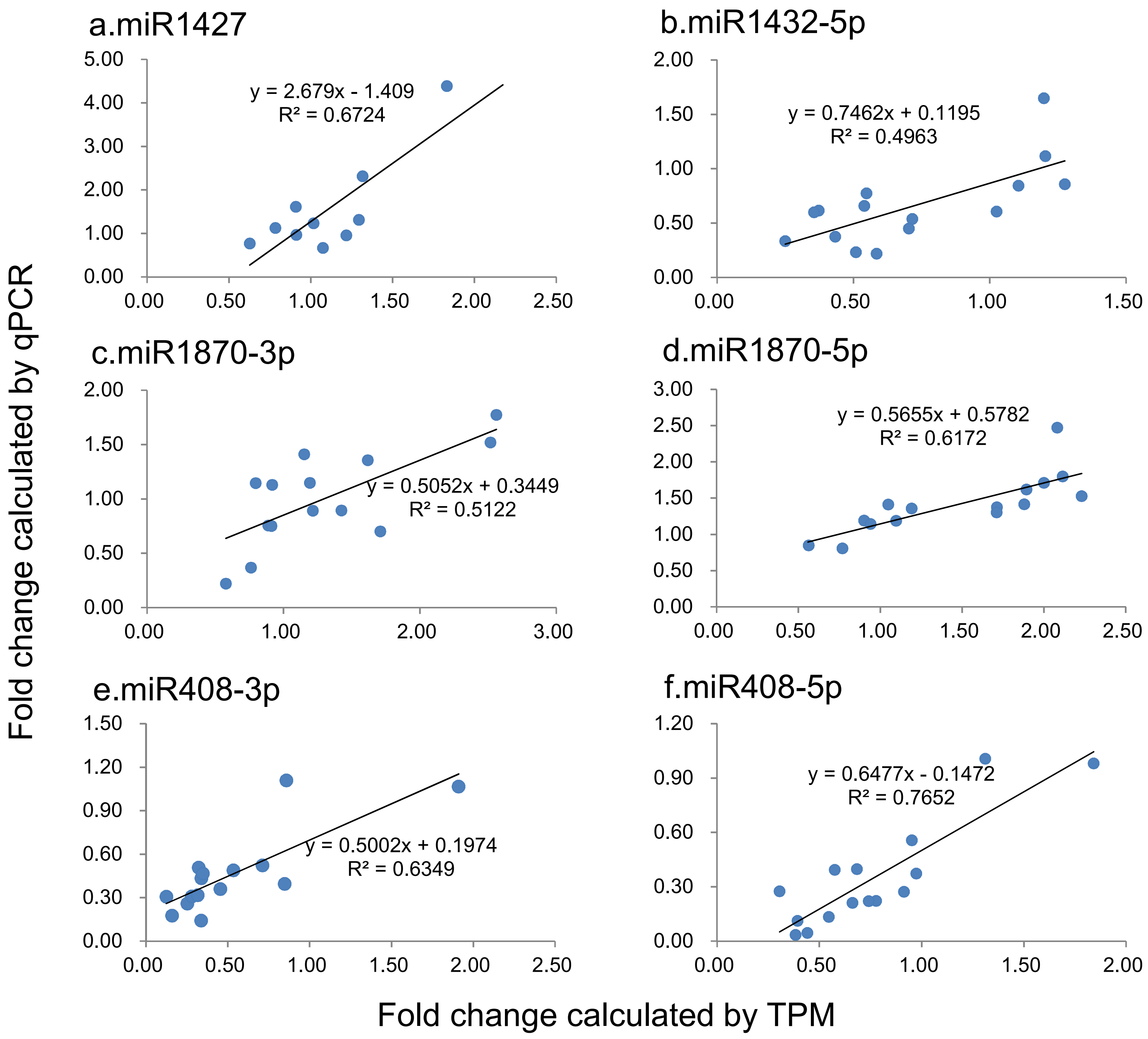
a.miR1427
y = 2.679x - 1.409
R² = 0.6724
b.miR1432-5p
y = 0.7462x + 0.1195
R² = 0.4963
c.miR1870-3p
y = 0.5052x + 0.3449
R² = 0.5122
d.miR1870-5p
y = 0.5655x + 0.5782
R² = 0.6172
e.miR408-3p
y = 0.5002x + 0.1974
R² = 0.6349
f.miR408-5p
y = 0.6477x - 0.1472
R² = 0.7652
Fold change calculated by qPCR
Fold change calculated by TPM

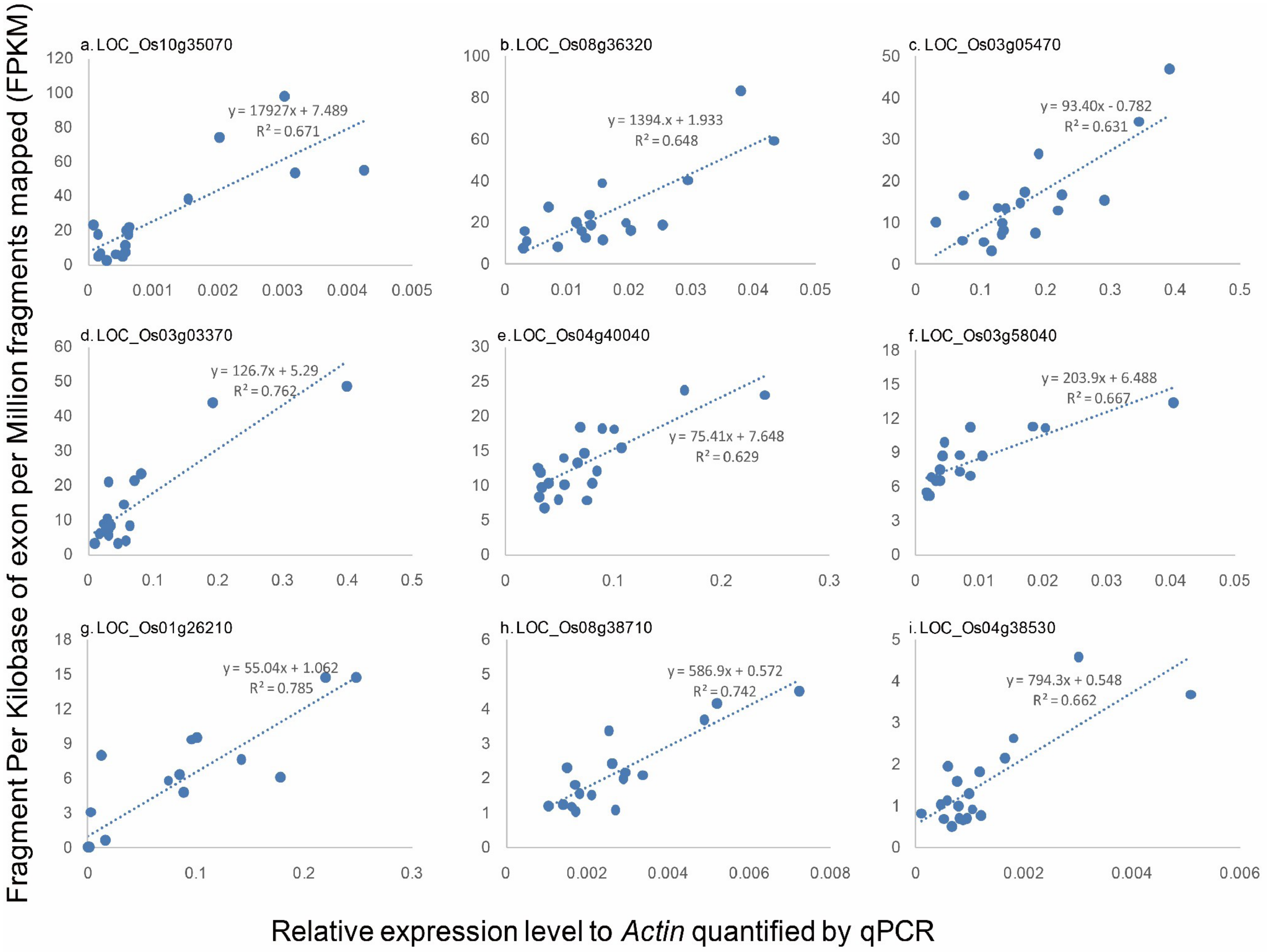
a. LOC_Os10g35070
y = 17927x + 7.489
R² = 0.671
b. LOC_Os08g36320
y = 1394.x + 1.933
R² = 0.648
c. LOC_Os03g05470
y = 93.40x - 0.782
R² = 0.631
d. LOC_Os03g03370
y = 126.7x + 5.29
R² = 0.762
e. LOC_Os04g40040
y = 75.41x + 7.648
R² = 0.629
f. LOC_Os03g58040
y = 203.9x + 6.488
R² = 0.667
g. LOC_Os01g26210
y = 55.04x + 1.062
R² = 0.785
h. LOC_Os08g38710
y = 586.9x + 0.572
R² = 0.742
i. LOC_Os04g38530
y = 794.3x + 0.548
R² = 0.662
Fragment Per Kilobase of exon per Million fragments mapped (FPKM)
Relative expression level to Actin quantified by qPCR
